# Correction: Ocean acidification at a coastal CO_2_ vent induces expression of stress-related transcripts and transposable elements in the sea anemone *Anemonia viridis*

**DOI:** 10.1371/journal.pone.0218009

**Published:** 2019-06-04

**Authors:** The PLOS ONE Staff

## Notice of republication

An incomplete, earlier version of this article was published in error. The publisher apologizes for the error. This article was republished on May 21, 2019 to correct for this error. Please download the article again to view the correct version. The originally published, uncorrected article and the republished, corrected article are provided here for reference.

## Supporting information

S1 File Originally published, uncorrected article. (PDF)

S2 File Republished, corrected article. (PDF)
