# Correction: Ocean acidification at a coastal CO_2_ vent induces expression of stress-related transcripts and transposable elements in the sea anemone *Anemonia viridis*

**DOI:** 10.1371/journal.pone.0230397

**Published:** 2020-03-10

**Authors:** The PLOS ONE Staff

A low-resolution version of Fig 3 was published in error. The authors have provided a higher resolution version for this Correction. The publisher apologizes for this error. Please see the updated Fig 3 here.

**Fig 3:**
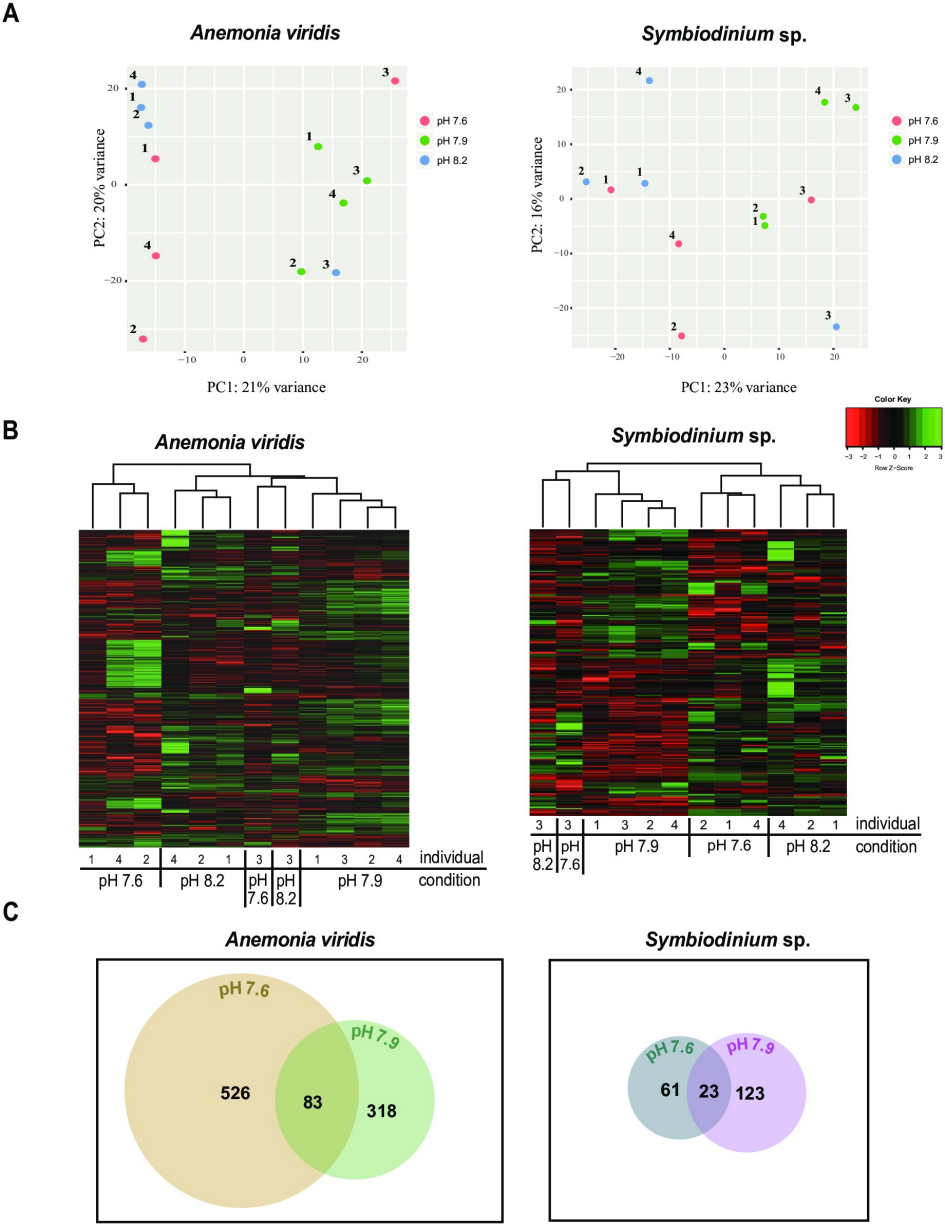
Differential gene expression profiles of *Anemonia viridis* and *Symbiodinium* sp. at the sampling sites. Differential expression (DE) pipeline using a glm edgeR approach was applied to account for both the day of sampling and the different pH where samples were taken. The DE analysis was performed separately for *A*. *viridis* and *Symbiodinium* sp. (A) Principal component analysis (PCA) plots show clustering similarity of individual samples. Numbers in the PCA plots represent different individuals sequenced. (B) Shown are heatmaps with hierarchically clustered, significantly differentially expressed (DE) transcripts between the sampling sites separately for *A*. *viridis* and *Symbiodinium* sp. (C) Venn diagrams visualize the number of private and shared DE-transcripts at pH 7.6 and pH 7.9 compared to normal conditions (pH 8.2). *A*. *viridis* contained 526 private DE-transcripts at pH 7.6 and 318 private DE-transcripts at pH 7.9. The symbiont contained 61 and 123 private DE-transcripts at pH 7.6 and pH 7.9, respectively. Venn diagrams were created using venneuler in R software.

## References

[1] Urbarova I, Forêt S, Dahl M, Emblem Å, Milazzo M, Hall-Spencer JM, et al (2019) Ocean acidification at a coastal CO_2_ vent induces expression of stress-related transcripts and transposable elements in the sea anemone *Anemonia viridis*. PLoS ONE 14(5): e0210358 10.1371/journal.pone.0210358 31067218 PMC6505742

