# Supplementary figures and images for: Ocean acidification at a coastal CO2 vent induces expression of stress-related transcripts and transposable elements in the sea anemone Anemonia viridis
Source: PLoS One. 2019 May 8;14(5):e0210358. doi: 10.1371/journal.pone.0210358 (PMC6505742; doi:10.1371/journal.pone.0210358)

S1 Fig. Gene expression profiles of *Anemonia viridis* and *Symbiodinium* sp. at low pH.

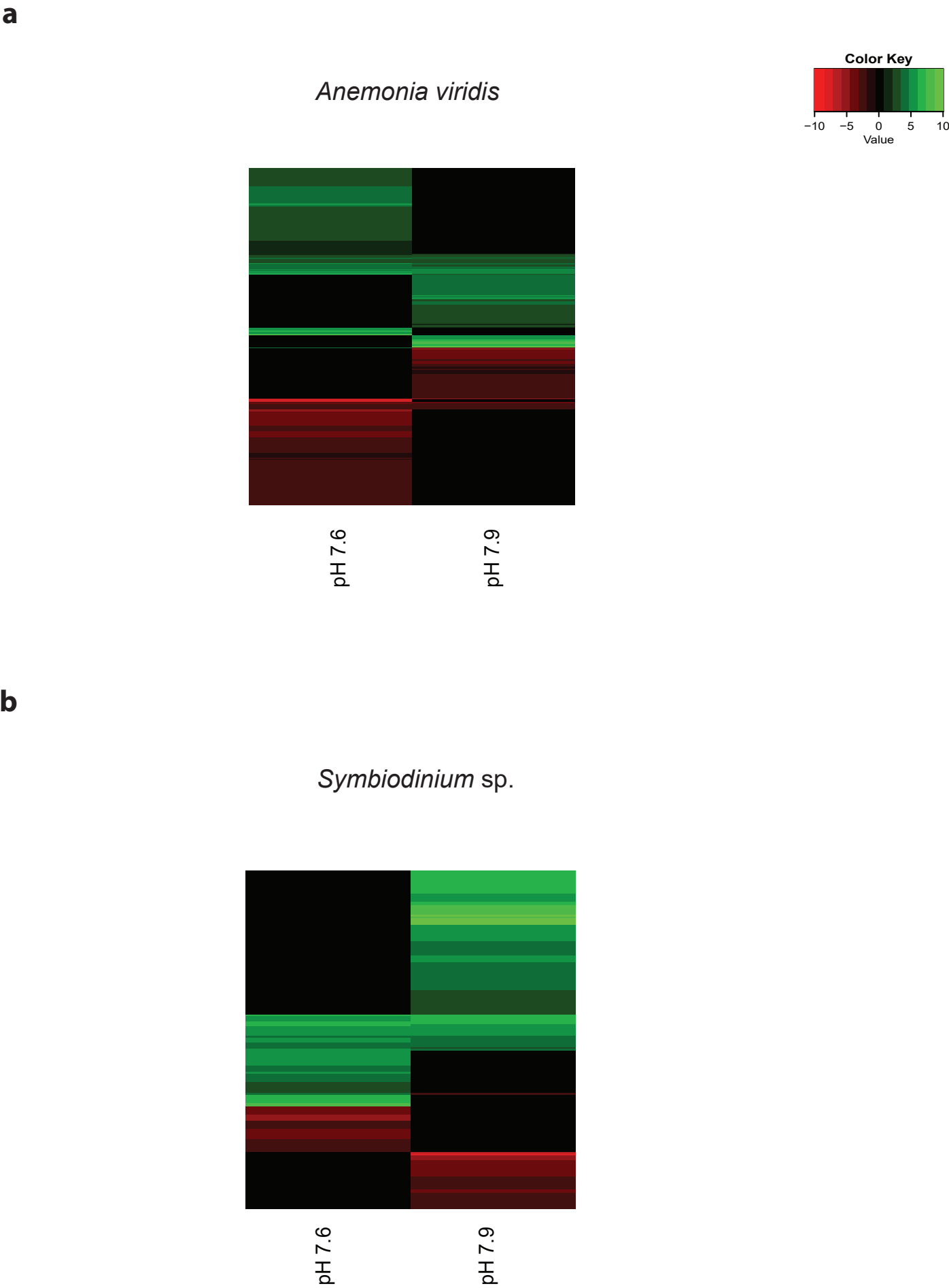

Supplement: S1 Fig — Shown are differential gene expression profiles of (A) the host and (B) the symbiont from two sampling locations of decreasing pH (pH 7.6 and pH 7.9) compared to normal seawater pH 8.2. The differential expression values are plotted as logarithmic fold change (logFC) values, and were calculated from the average expression values of four individuals per sampling location. The heatmap serves only as a visualization of transcripts that are significantly up- and down-regulated at the two low pH conditions compared to normal seawater pH 8.2. (PDF) [file pone.0210358.s001.pdf]

**S3 Fig. Expression levels of six candidate reference genes at the sampling sites.**

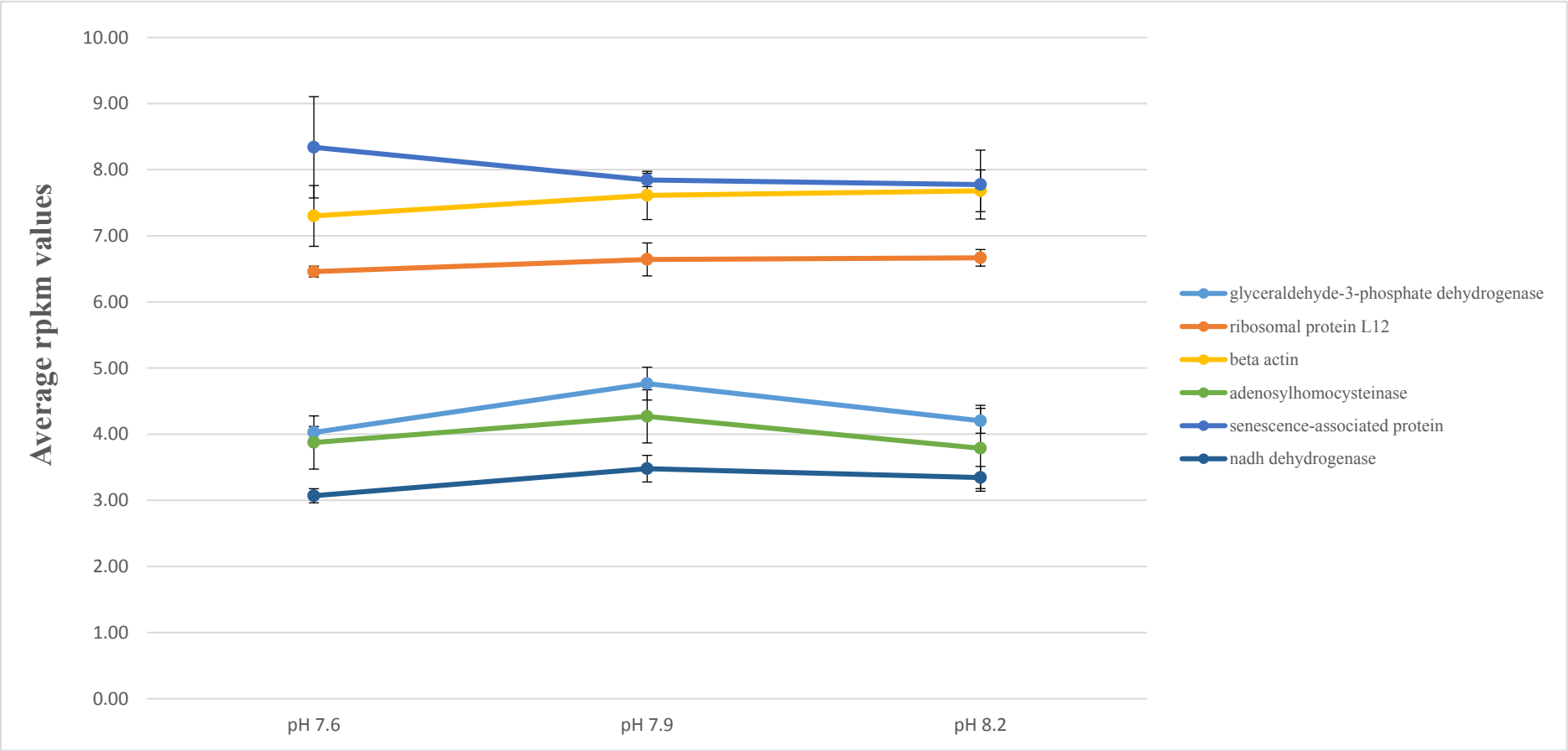

Supplement: S3 Fig — The expression levels of six potential reference transcripts: glyceraldehyde 3-phosphate dehydrogenase (GAPDH), ribosomal protein L12 (RPL12), beta-actin, adenosylhomocysteinase (AHCY), senescence-associated protein and NADH dehydrogenase (NDH), were assessed among the different sampling sites. Presented are the normalized values of these transcripts from the RNA-seq transcript expression matrix. Three transcripts were successfully established as reference transcripts after qPCR testing: GAPDH, RPL12 and beta-actin. (PDF) [file pone.0210358.s003.pdf]
