# Supplementary material for: Ocean acidification at a coastal CO2 vent induces expression of stress-related transcripts and transposable elements in the sea anemone Anemonia viridis
Source: PLoS One. 2019 May 8;14(5):e0210358. doi: 10.1371/journal.pone.0210358 (PMC6505742; doi:10.1371/journal.pone.0210358)
Supplement: S2 Fig — We assessed the expression of nine transcripts reported as differentially expressed in our RNA-seq analysis by quantitative (q)PCR. Three individuals per each condition studied (pH 7.6, pH 7.9 and pH 8.2) were examined, and values were normalized to the expression of our reference transcripts: ribosomal protein L12 (RPL12), beta-actin and glyceraldehyde 3-phosphate (GAPDH). Gene expression at two low seawater pH sites (pH 7.6 and pH 7.9) is shown as a relative measure with standard deviations compared to the gene expression at normal seawater pH 8.2. (PDF) [file pone.0210358.s002.pdf]

S2 Fig. Verification of significantly differentially expressed genes by quantitative PCR.

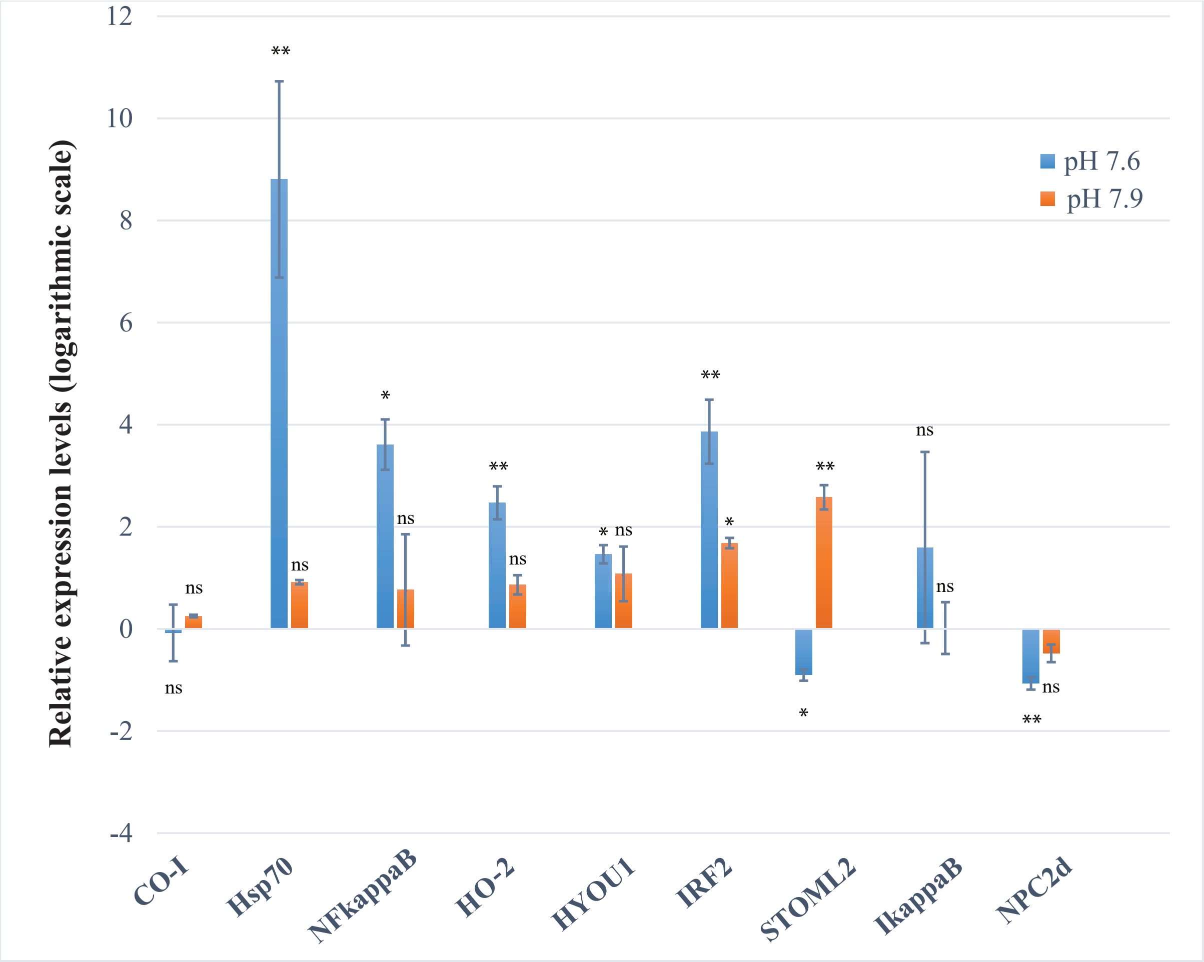

One-Way ANOVA test with Bonferroni's Multiple Comparison correction was performed on the whole data set. Results are indicated in the figure as follows:

** - significantly differential expression compared to control pH 8.2 ($p < 0.01$)

* - significantly differential expression compared to control pH 8.2 ($p < 0.05$)

ns - non-significant differential expression compared to control pH 8.2 ($p > 0.05$)

CO-I - cytochrome c oxidase subunit 1, Hsp70 - heat shock protein 70, NFkappaB - nuclear factor kappa B, HO-2 - heme oxygenase 2-like, HYOU1 - hypoxia upregulated protein 1, IFR2 - interferon regulatory protein 2, STOML2 - mitochondrial stomatin protein 2-like, IkappaB – inhibitor of NF-kappaB, NPC2d – Niemann-Pick type C2-like protein (variant d)
