# Supplementary material for: Ocean acidification at a coastal CO2 vent induces expression of stress-related transcripts and transposable elements in the sea anemone Anemonia viridis
Source: PLoS One. 2019 May 8;14(5):e0210358. doi: 10.1371/journal.pone.0210358 (PMC6505742; doi:10.1371/journal.pone.0210358)

S1 Table. Sequencing information and metrics of the assembled reference transcriptome of *Anemonia viridis*.

Raw reads recovered from transcriptome sequencing on the Ion Torrent PGM platform

Individuals [1]	Number of raw reads	Reads removed [%]	Reads aligned to reference [%]
pH 7.6 - 1	1 954 219	12.5	93.9
pH 7.6 - 2	1 924 941	8.3	93.6
pH 7.6 - 3	2 001 386	10.4	96.3
pH 7.6 - 4	1 675 136	9.0	95.1
pH 7.9 - 1	1 792 393	11.3	93.2
pH 7.9 - 2	2 014 437	9.6	93.4
pH 7.9 - 3	2 044 262	11.4	96.4
pH 7.9 - 4	2 418 930	7.7	97.0
pH 8.2 - 1	1 805 994	10.5	94.0
pH 8.2 - 2	1 975 702	9.2	92.2
pH 8.2 - 3	1 772 784	8.8	96.4
pH 8.2 - 4	3 510 099	9.9	97.2

[1] *Four individuals from each condition were analysed and several barcoded transcriptome libraries were sequenced together on 318 v2 PGM chips. All sequences coming from one individual from the same sequencing library were pooled before trimming and quality filtering.*

Metrics of the assembled reference transcriptome of *Anemonia viridis* [1]

**Number of sequences**	**154,015**
**Smallest contig**	221
**Largest contig**	8,648
**Number of bases**	82,023,224
**Mean length**	532,6
**Number of contigs over 1k**	15,150
**Number of contigs over 10k**	0
**Number of contigs with orf**	36,519
**N90**	280
**N70**	404
**N50**	599
**N30**	956
**N10**	1,770
**GC content**	47,4%

[1] *Shown is a distribution of contig lengths in the assembly and several other metrics for the reference assembly created using R and transcriptome assembly quality analysis software transrate (Smith-Unna, R et al., Genome Res 2016, 26:1134-1144).*

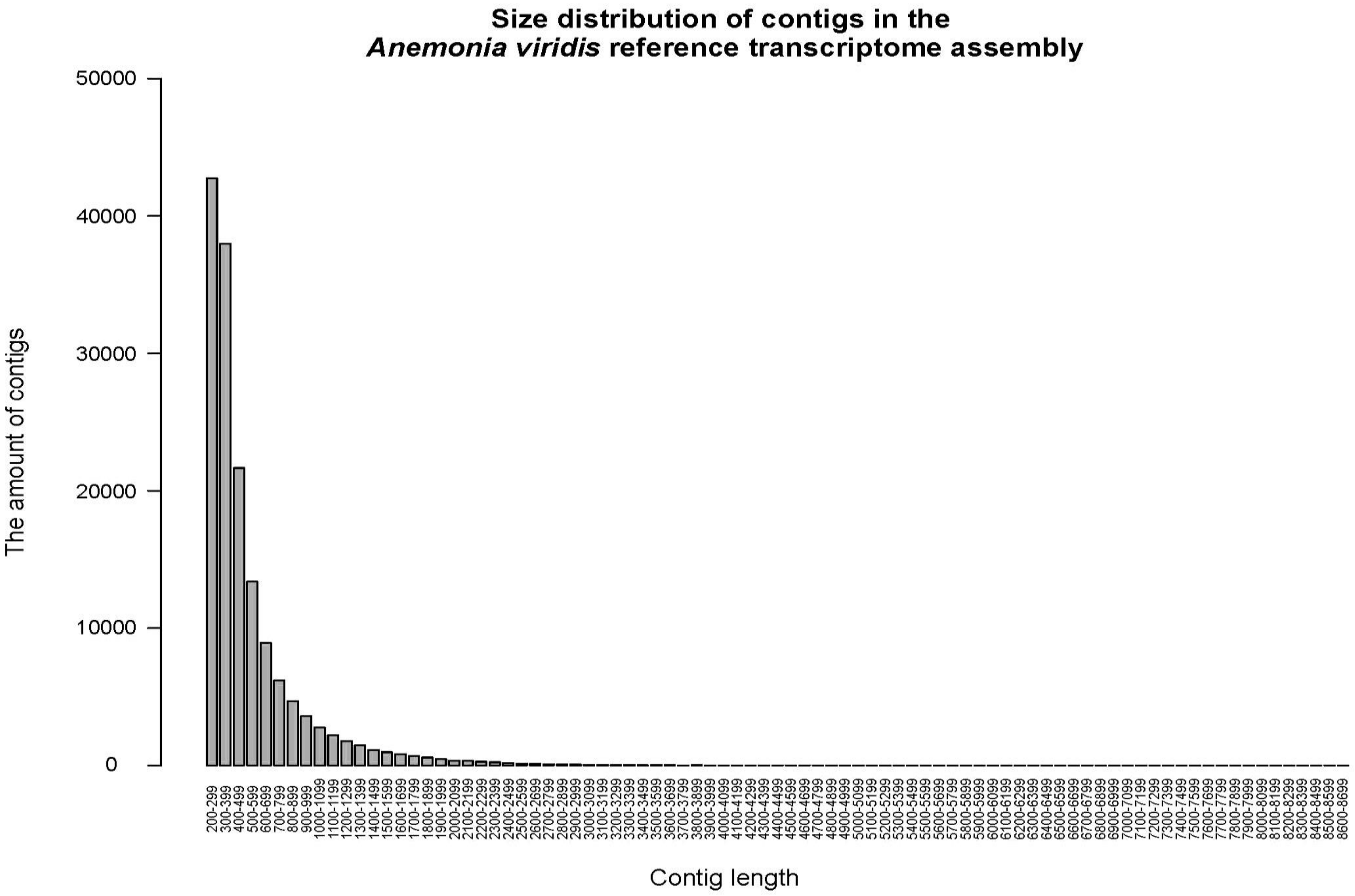

There are 27,273 published and predicted protein gene sequences from the sea anemone *N. vectensis* (*Putnam, NH et al., Science 2007, 317: 86-94*) and 47,014 predicted protein gene sequences from the symbiont *Symbiodinium minutum* (*Shoguchi, E et al., Curr Biol* 2013, 23: 1399-1408).

In our analysis, we obtained 90,535 *A. viridis* contigs and 63,480 symbiont contigs.

Using reciprocal BLAST, 41,495 (45.7%) *A. viridis* contigs had hits in the predicted protein gene sequences from the sea anemone *N. vectensis* (e-value=10^{-3}). 22,554 (82.7%) predicted protein gene sequences from the sea anemone *N. vectensis* could be found in *A. viridis* contigs (e-value=10^{-3}).

For the symbiont, 45,625 (71.7%) contigs had hits predicted protein gene sequences from the symbiont *Symbiodinium minutum* (e-value=10^{-3}). 33,500 (71.3%) predicted protein gene sequences from the symbiont *Symbiodinium minutum* could be found in symbiont contigs (e-value=10^{-3}).

HOST TRANSCRIPTOME

**Number of sequences**	**90,535**
**Smallest contig**	224
**Largest contig**	8,648
**Number of bases**	48,247,078
**Mean length**	532,9
**Number of contigs over 1k**	9,103
**Number of contigs over 10k**	0
**Number of contigs with orf**	16,272
**N90**	277
**N70**	396
**N50**	595
**N30**	1,002
**N10**	1,923
**GC content**	40,8%

SYMBIONT TRANSCRIPTOME

**Number of sequences**	**63,480**
**Smallest contig**	224
**Largest contig**	4,249
**Number of bases**	33,776,545
**Mean length**	532,1
**Number of contigs over 1k**	6,047
**Number of contigs over 10k**	0
**Number of contigs with orf**	20,247
**N90**	284
**N70**	415
**N50**	603
**N30**	911
**N10**	1,551
**GC content**	56,9%

To test transcriptome completeness, we ran our transcriptome assembly using BUSCO v3.0.2 (*Simão FA et al., Bioinformatics 2015, 31:3210-3212*), separately for the host and the symbiont. For the host, we were able to detect 96% of 978 metazoan single-copy orthologous genes (676 complete and 263 fragmented). For the symbiont, we detected 63.4% of 303 eukaryote single-copy orthologous genes (94 complete and 98 fragmented). Relatively low recovery of conserved eukaryote genes in *Symbiodinium* was not surprising, since this has been observed before when BUSCO was run at default settings (*Liu et al., Commun Biol. 2018; 1: 95, Burns et al., eLife 2017, 6:e22054*). It seems that the relative incompleteness of the transcriptome does not affect inference of differentially expressed genes (*Burns et al., eLife 2017, 6:e22054*). In addition, we also checked later on for presence of symbiont stress-response genes in the symbiont transcriptome assembly (S10 Table). These were not found as differentially expressed in our study.

PSyTrans software tool performance

We identified relatively high amount of hits when performing inter-changed alignments (host-reads to symbiont transcripts and symbiont-reads to host-transcripts). However, host-reads always had higher sequence similarity to host transcripts than to symbiont transcripts. To further test the performance of the PSyTrans software tool, we downloaded an aposymbiotic sea anemone *Calliactis polypus* (SRR3205709) and pure symbiont culture of *Symbiodinium* sp. (SRR1300263) and assembled these sequences *de novo* using Trinity to gain a mixed *C. polypus* and *Symbiodinium* sp. transcriptome. This assembled transcriptome was run through the PSyTranS tool; see below the figure of the separation of host and symbiont contigs. From the initial mixed assembly of 283,390 contigs, we gained 147,987 host contigs with average GC content 38% and 135,403 symbiont contigs with average GC content 59%. Additionally, we verified that the host contigs separated by the PSyTranS tool were correctly associated with *C. polypus* by BLASTing them to a *de novo* assembly of *C. polypus* reads alone. The same verification analysis was also performed for symbiont contigs, in addition to inter-changed alignments. These analyses confirmed that the contigs from the mixed assembly were correctly BLASTing to their corresponding *de novo* assemblies of the species reads alone, therefore correctly matching the species origin in the mixed assembly.

Separation of *C. polypus* and *Symbiondinium* sp.
mixed *de novo* assembled transcriptome

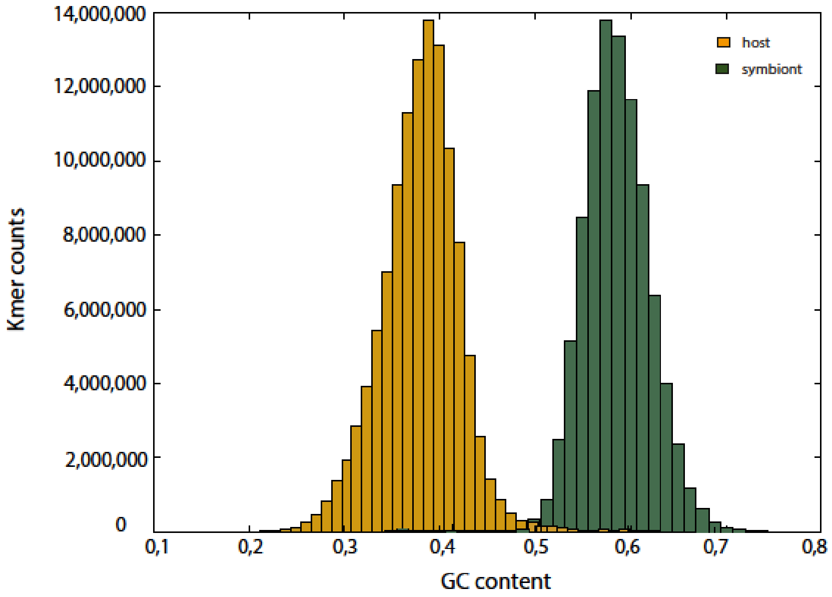

Supplement: S1 Table — (PDF) [file pone.0210358.s004.pdf]
