# Supplementary material for: Ocean acidification at a coastal CO2 vent induces expression of stress-related transcripts and transposable elements in the sea anemone Anemonia viridis
Source: PLoS One. 2019 May 8;14(5):e0210358. doi: 10.1371/journal.pone.0210358 (PMC6505742; doi:10.1371/journal.pone.0210358)
Supplement: S2 Table — Shown are primer sequences used for PCR amplification of symbiont-specific nuclear apx gene, together with 12 primer pairs used for verification of results from differential expression analysis by qPCR. (PDF) [file pone.0210358.s005.pdf]

S2 Table. Primer pairs used for regular and quantitative PCR analysis.

**Transcript** [1]	**Primers used** [2]
**Ascorbate peroxidase (APX)**	**FP** CGTCCAGGACCTCGAGAACT<br>**RP** TTGAGCTCGCCATCAGAGAA
**beta-actin**	**FP** CTGATGGACAGGTCATCACCAT<br>**RP** CTCGTGGATACCAGCAGATTCC
**Ribosomal protein L12 (RPL12)**	**FP** GCTTTGAAGGAACCACCAAG<br>**RP** TCTTGGCAATGTCAACAAGC
**Glyceraldehyde 3-phosphate dehydrogenase (GAPDH)**	**FP** CAAGGCAGTTGGTAGTGCAA<br>**RP** TGCACCCATGTTTGTAATGG
**Heat shock protein 70 (Hsp70)**	**FP** GAAAAAGCTCTCCGTGATGC<br>**RP** AAGCTCTGGACCTTGGGAAT
**Heme oxygenase 2-like (HO-2)**	**FP** CGTACTCTTTGGTTGCCTCTG<br>**RP** ACCCACATGAACTGGAAAGG
**Interferon regulatory factor 2 (IRF2)**	**FP** TCTGAATTCCCAGGGATGAG<br>**RP** TCTCGTTCCTTGTCCCAAGT
**NF-kappaB**	**FP** TGAAGGACCATCACATGGAG<br>**RP** ACATGGCCCACGATAGTTGT
**hypoxia up-regulated protein 1-like (HYOU1)**	**FP** GATTAGCGGCCTGGTAAACA<br>**RP** CGTGTGCCAAAGGTACAAGA
**Stomatin-like protein 2 (STOML2)**	**FP** AAAAGCAAAGGCCAGATCAA<br>**RP** CTCTGCGACATTGAGACCTG
**Cytochrome c oxidase subunit I (CO-I)**	**FP** ATAGAAGACGCACCCGCTAA<br>**RP** TTATCCTCCTCTTTCTGGCATT
**I-kappaB**	**FP** TCCATGACGATCAACTGCAT<br>**RP** AGACACCACTCCACCTTGCT
**Niemann-Pick C2-like protein, variant d (NPC2d)**	**FP** AACGTTCGTCGTTTTGGAGT<br>**RP** GGCATTTTAGCCCGTATCCT

[1] *Contig best blast hit*

[2] *FP – forward primer, RP – reverse primer*
