# Supplementary material for: Ocean acidification at a coastal CO2 vent induces expression of stress-related transcripts and transposable elements in the sea anemone Anemonia viridis
Source: PLoS One. 2019 May 8;14(5):e0210358. doi: 10.1371/journal.pone.0210358 (PMC6505742; doi:10.1371/journal.pone.0210358)
Supplement: S3 Table — Shown is the R script of glm edgeR analyses that was used to find differentially expressed genes between the various pH conditions separately for the host and for the symbiont, using four different individuals per condition. Additionally, PCA plots and heatmaps were created. (PDF) [file pone.0210358.s006.pdf]

S3 Table. R script for differential expression analyses.

```
#inputting the count files, creating DGE file and performing filtering
library(limma)
library(edgeR)
setwd("F:/abundance_estimation_new/bwa_express")
files_bwa <- c("ph7.6_1.xprs","ph7.6_2.xprs","ph7.6_3.xprs","ph7.6_4.xprs",
               "ph7.9_1.xprs","ph7.9_2.xprs","ph7.9_3.xprs","ph7.9_4.xprs",
               "ph8.2_1.xprs","ph8.2_2.xprs","ph8.2_3.xprs","ph8.2_4.xprs")
group <- factor(c(1,1,1,1,2,2,2,2,3,3,3,3))
DE_bwa_col5 <- readDGE(files_bwa, path=NULL, columns=c(2, 5), group=group, labels=NULL)
minCounts     <- 10
minConditions <- 4
DE_bwa_col5$counts <- DE_bwa_col5$counts[apply(DE_bwa_col5$counts >= minCounts, 1, sum) >= minConditions, ]

#separating host transcripts
hostNames   <- scan('../hostNames_ITpooled_20150526_cd-hit-est_0.90.txt', what='character')
hostCounts  <- DE_bwa_col5[rownames(DE_bwa_col5) %in% hostNames, ]

#creating PCA plot
library(DESeq2)
x     <- as.data.frame(hostCounts$counts)
y     <- as.data.frame(designMatrix)
dds   <- DESeqDataSetFromMatrix(countData = x, colData=y, design=~condition)
dds   <- DESeq(dds)
rld   <- rlogTransformation(dds)
library(ggplot2)
p     <- plotPCA(rld, intgroup = c("condition"), ntop = 1000)
p     <- p + geom_text(aes_string(x = "PC1", y = "PC2", label = "name"), color = "black")
print(p)

#running glm approach, accounting for two conditions in the model - pH and day of sampling
diff_pH <-
factor(c("ph7.6","ph7.6","ph7.6","ph7.6","ph7.9","ph7.9","ph7.9","ph7.9","ph8.2","ph8.2","ph8.2","ph8.2"))
day <- factor(c("13/5","14/5","13/5","14/5","13/5","14/5","13/5","14/5","13/5","14/5","13/5","14/5"))
hostCounts_df <- data.frame(Sample=colnames(hostCounts),diff_pH,day)
design        <- model.matrix(~day + diff_pH, data=hostCounts_df)
hostCounts <- estimateGLMCommonDisp(hostCounts, design, verbose=TRUE)
hostCounts <- estimateGLMTrendedDisp(hostCounts, design, method ="power")
```

```
hostCounts <- estimateGLMTagwiseDisp(hostCounts, design)

#extracting significantly differentially expressed genes for host
ExactTest7.6to8.2 <- exactTest(hostCounts, pair=c("1","3"))
ExactTest7.9to8.2 <- exactTest(hostCounts, pair=c("2","3"))
ExactTest7.6to7.9 <- exactTest(hostCounts, pair=c("1","2"))
topTags_7.6to8.2  <- topTags(ExactTest7.6to8.2, n=nrow(ExactTest7.6to8.2$table))
topTags_7.9to8.2  <- topTags(ExactTest7.9to8.2, n=nrow(ExactTest7.9to8.2$table))
topTags_7.6to7.9  <- topTags(ExactTest7.6to7.9, n=nrow(ExactTest7.6to7.9$table))

#creating heatmap for host
sizes              <- read.table(file="F:/goseq/Avir_ITpooled_NewlyExported_20150526_justLength.txt",
                       sep="\t", header=TRUE)
ordered.gene.length <- sizes$seq_length[match(rownames(hostCounts$counts),sizes$genes)]
hostCounts_rpkm     <- rpkm(hostCounts, normalized.lib.sizes=TRUE, log=TRUE,
                       prior.count=0.25,gene.length=ordered.gene.length)

host_filtered_7.6to8.2 <- hostCounts_rpkm[rownames(topTags_7.6to8.2$table)[topTags_7.6to8.2$table$FDR<0.05],]
host_filtered_7.9to8.2 <- hostCounts_rpkm[rownames(topTags_7.9to8.2$table)[topTags_7.9to8.2$table$FDR<0.05],]
host_filtered_7.6to7.9 <- hostCounts_rpkm[rownames(topTags_7.6to7.9$table)[topTags_7.6to7.9$table$FDR<0.05],]

names_7.6to8.2 = rownames(host_filtered_7.6to8.2)
names_7.9to8.2 = rownames(host_filtered_7.9to8.2)
names_7.6to7.9 = rownames(host_filtered_7.6to7.9)
two_names      = union(names_7.6to8.2, names_7.9to8.2)
allnames       = union(two_names,names_7.6to7.9)
hostCounts_filtered_rpkm = hostCounts_rpkm[allnames, ]

library(gplots)
colnames(hostCounts_filtered_rpkm) <- c("7.6_1", "7.6_2", "7.6_3","7.6_4", "7.9_1", "7.9_2", "7.9_3", "7.9_4",
"8.2_1", "8.2_2", "8.2_3", "8.2_4")
heatmap.2(as.matrix(hostCounts_filtered_rpkm), col=redgreen, density.info="none",
          key=TRUE, keysize=1.2, trace="none", margins = c(5,5), dendrogram="column", labRow=FALSE,
          scale="row", cexRow=0.2, cexCol=1.5, symkey=FALSE, main = "Anemonia exposed to low pH")

#extraction of up- and downregulated genes - 7.6vs8.2
de1         <- decideTestsDGE(ExactTest7.6to8.2)
#upregulated genes
geneList1   <- which(de1[,1] == -1)
#downregulated genes
```

```
geneList2   <- which(de1[,1] == 1)
#get the expression matrix
geneNames1  <- rownames(hostCounts$counts[geneList1,])
expVal1     <- hostCounts_filtered_rpkm[rownames(hostCounts_filtered_rpkm) %in% geneNames1, ]
geneNames2  <- rownames(hostCounts$counts[geneList2,])
expVal2     <- hostCounts_filtered_rpkm[rownames(hostCounts_filtered_rpkm) %in% geneNames2, ]
geneNames12 <- c(geneNames1, geneNames2)
expVal12    <- hostCounts_filtered_rpkm[rownames(hostCounts_filtered_rpkm) %in% geneNames12, ]

#extraction of up- and downregulated genes - 7.9vs8.2
de2         <- decideTestsDGE(ExactTest7.9to8.2)
#upregulated genes
geneList3   <- which(de2[,1] == -1)
#downregulated genes
geneList4   <- which(de2[,1] == 1)
#get the expression matrix
expVal3     <- (hostCounts$counts[geneList3,])
expVal4     <- (hostCounts$counts[geneList4,])
geneNames3  <- rownames(expVal3)
geneNames4  <- rownames(expVal4)
geneNames34 <- c(geneNames3, geneNames4)

#extraction of up- and downregulated genes - 7.6vs7.9
de3         <- decideTestsDGE(ExactTest7.6to7.9)
#upregulated genes
geneList5   <- which(de3[,1] == -1)
#downregulated genes
geneList6   <- which(de3[,1] == 1)
#get the expression matrix
expVal5     <- (hostCounts$counts[geneList5,])
expVal6     <- (hostCounts$counts[geneList6,])
geneNames5  <- rownames(expVal5)
geneNames6  <- rownames(expVal6)
geneNames56 <- c(geneNames5, geneNames6)

#pipeline for symbiont differential gene expression analysis
setwd("F:/abundance_estimation_new/bwa_express")
files_bwa <- c("ph7.6_1.xprs","ph7.6_2.xprs","ph7.6_3.xprs","ph7.6_4.xprs",
               "ph7.9_1.xprs","ph7.9_2.xprs","ph7.9_3.xprs","ph7.9_4.xprs",
               "ph8.2_1.xprs","ph8.2_2.xprs","ph8.2_3.xprs","ph8.2_4.xprs")
```

```
group <- factor(c(1,1,1,1,2,2,2,2,3,3,3,3))
DE_bwa_col5 <- readDGE(files_bwa, path=NULL, columns=c(2, 5), group=group, labels=NULL)
minCounts     <- 10
minConditions <- 4
DE_bwa_col5$counts <- DE_bwa_col5$counts[apply(DE_bwa_col5$counts >= minCounts, 1, sum) >= minConditions, ]

#separating symbiont transcripts
symbNames   <- scan('../symbNames_ITpooled_20150526_cd-hit-est_0.90.txt', what='character')
symbCounts  <- DE_bwa_col5[rownames(DE_bwa_col5) %in% symbNames, ]

#creating PCA plot
library(DESeq2)
x_s         <- as.data.frame(symbCounts$counts)
y_s         <- as.data.frame(designMatrix)
dds_s       <- DESeqDataSetFromMatrix(countData = x_s, colData=y_s, design=~condition)
dds_s       <- DESeq(dds_s)
rld_s       <- rlogTransformation(dds_s)
library(ggplot2)
p_s         <- plotPCA(rld_s, intgroup = c("condition"), ntop = 1000)
p_s         <- p_s + geom_text(aes_string(x = "PC1", y = "PC2", label = "name"), color = "black")
print(p_s)

#running glm approach, accounting for two conditions in the model - pH and day of sampling
diff_pH <-
factor(c("ph7.6","ph7.6","ph7.6","ph7.6","ph7.9","ph7.9","ph7.9","ph7.9","ph8.2","ph8.2","ph8.2","ph8.2"))
day <- factor(c("13/5","14/5","13/5","14/5","13/5","14/5","13/5","14/5","13/5","14/5","13/5","14/5"))
symbCounts_df <- data.frame(Sample=colnames(symbCounts),diff_pH,day)
design      <- model.matrix(~day + diff_pH, data=symbCounts_df)
rownames(design) <- colnames(symbCounts_df)
symbCounts <- estimateGLMCommonDisp(symbCounts, design, verbose=TRUE)
symbCounts <- estimateGLMTrendedDisp(symbCounts, design, method="power")
symbCounts <- estimateGLMTagwiseDisp(symbCounts, design)

#extracting significantly differentially expressed genes for symbiont
ExactTest7.6to8.2s <- exactTest(symbCounts, pair=c("1","3"))
ExactTest7.9to8.2s <- exactTest(symbCounts, pair=c("2","3"))
ExactTest7.6to7.9s <- exactTest(symbCounts, pair=c("1","2"))
topTags_7.6to8.2s  <- topTags(ExactTest7.6to8.2s, n=nrow(ExactTest7.6to8.2s$table))
topTags_7.9to8.2s <- topTags(ExactTest7.9to8.2s, n=nrow(ExactTest7.9to8.2s$table))
topTags_7.6to7.9s <- topTags(ExactTest7.6to7.9s, n=nrow(ExactTest7.6to7.9s$table))
```

```
#creating heatmap for symbiont
sizes        <- read.table(file="F:/Avir_ITpooled_NewlyExported_20150526_justLength.txt", sep="\t", header=TRUE)
ordered.gene.length <- sizes$seq_length[match(rownames(symbCounts$counts),sizes$genes)]
symbCounts_rpkm     <- rpkm(symbCounts, normalized.lib.sizes=TRUE, log=TRUE,
prior.count=0.25,gene.length=ordered.gene.length)
symb_filtered_7.6to8.2 <- symbCounts_rpkm[rownames(topTags_7.6to8.2s$table)[topTags_7.6to8.2s$table$FDR<0.05],]
symb_filtered_7.9to8.2 <- symbCounts_rpkm[rownames(topTags_7.9to8.2s$table)[topTags_7.9to8.2s$table$FDR<0.05],]
symb_filtered_7.6to7.9 <- symbCounts_rpkm[rownames(topTags_7.6to7.9s$table)[topTags_7.6to7.9s$table$FDR<0.05],]

names_7.6to8.2s = rownames(symb_filtered_7.6to8.2)
names_7.9to8.2s = rownames(symb_filtered_7.9to8.2)
names_7.6to7.9s = rownames(symb_filtered_7.6to7.9)
two_names_symb  = union(names_7.6to8.2s, names_7.9to8.2s)
allnames_symb   = union(two_names_symb,names_7.6to7.9s)
symbCounts_filtered_rpkm = symbCounts_rpkm[allnames_symb, ]

library(gplots)
colnames(symbCounts_filtered_rpkm) <- c("7.6_1", "7.6_2", "7.6_3", "7.6_4", "7.9_1", "7.9_2", "7.9_3", "7.9_4",
"8.2_1", "8.2_2", "8.2_3","8.2_4")
heatmap.2(as.matrix(symbCounts_filtered_rpkm), col=redgreen, density.info="none", trace="none",
          margins = c(5,5), dendrogram="column", symbreaks=FALSE, scale='row', key=TRUE, keysize=1.2,
          cexCol=1.5, labRow=NA, symkey=FALSE, main = "Symbiodinium exposed to low pH")

#extraction of up- and downregulated genes - 7.6vs8.2
de1s         <- decideTestsDGE(ExactTest7.6to8.2s)
#upregulated genes
geneList1s   <- which(de1s[,1] == -1)
#downregulated genes
geneList2s   <- which(de1s[,1] == 1)
#get the expression matrix
geneNames1s  <- rownames(symbCounts$counts[geneList1s,])
expVal1s     <- symbCounts_filtered_rpkm[rownames(symbCounts_filtered_rpkm) %in% geneNames1s, ]
geneNames2s  <- rownames(symbCounts$counts[geneList2s,])
expVal2s     <- symbCounts_filtered_rpkm[rownames(symbCounts_filtered_rpkm) %in% geneNames2s, ]
geneNames12s <- c(geneNames1s, geneNames2s)
expVal12s    <- symbCounts_filtered_rpkm[rownames(symbCounts_filtered_rpkm) %in% geneNames12s, ]

#extraction of up- and downregulated genes - 7.9vs8.2
de2s         <- decideTestsDGE(ExactTest7.9to8.2s)
```

```
#upregulated genes
geneList3s   <- which(de2s[,1] == -1)
#downregulated genes
geneList4s   <- which(de2s[,1] == 1)
#get the expression matrix
geneNames3s  <- rownames(symbCounts$counts[geneList3s,])
geneNames4s  <- rownames(symbCounts$counts[geneList4s,])
geneNames34s <- c(geneNames3s, geneNames4s)
expVal3s     <- symbCounts_filtered_rpkm[rownames(symbCounts_filtered_rpkm) %in% geneNames3s, ]
expVal4s     <- symbCounts_filtered_rpkm[rownames(symbCounts_filtered_rpkm) %in% geneNames4s, ]

#extraction of up- and downregulated genes - 7.6vs7.9
de3s         <- decideTestsDGE(ExactTest7.6to7.9s)
#upregulated genes
geneList5s   <- which(de3s[,1] == -1)
#downregulated genes
geneList6s   <- which(de3s[,1] == 1)
#get the expression matrix
geneNames5s  <- rownames(symbCounts$counts[geneList5s,])
geneNames6s  <- rownames(symbCounts$counts[geneList6s,])
geneNames56s <- c(geneNames5s, geneNames6s)
expVal5s     <- symbCounts_filtered_rpkm[rownames(symbCounts_filtered_rpkm) %in% geneNames5s, ]
expVal6s     <- symbCounts_filtered_rpkm[rownames(symbCounts_filtered_rpkm) %in% geneNames6s, ]
```
