# Supplementary material for: Ocean acidification at a coastal CO2 vent induces expression of stress-related transcripts and transposable elements in the sea anemone Anemonia viridis
Source: PLoS One. 2019 May 8;14(5):e0210358. doi: 10.1371/journal.pone.0210358 (PMC6505742; doi:10.1371/journal.pone.0210358)
Supplement: S4 Table — Shown is the R script used to investigate in detail the influence of each variable on the differential gene expression analyses separately for the host and for the symbiont. Differentially expressed genes identified in our glm edgeR analysis were separated based on the influence of the two variables in our study, pH condition and day of sampling. By forming contrasts from the design matrix in the glm edgeR pipeline, we were able to identify 1124 DE-transcripts for the host Anemonia viridis (91.2%) and 249 DE-transcripts for the symbiont Symbiodinium sp. (85.6%) affected by the pH condition variable only. We therefore concluded that day of sampling did not have significant effect on the identification of the differentially expressed genes between the individual pH conditions. (PDF) [file pone.0210358.s007.pdf]

S4 Table. R script to show the influence of each variable in glm edgeR analyses.

```
#pipeline for the host - inputting the count files, creating DGE file and performing filtering
library(limma)
library(edgeR)
setwd("F:/abundance_estimation_new/bwa_express")
files_bwa <- c("ph7.6_1.xprs","ph7.6_2.xprs","ph7.6_3.xprs","ph7.6_4.xprs",
               "ph7.9_1.xprs","ph7.9_2.xprs","ph7.9_3.xprs","ph7.9_4.xprs",
               "ph8.2_1.xprs","ph8.2_2.xprs","ph8.2_3.xprs","ph8.2_4.xprs")
group <- factor(c(1,1,1,1,2,2,2,2,3,3,3,3))
DE_bwa_col5 <- readDGE(files_bwa, path=NULL, columns=c(2, 5), group=group, labels=NULL)
minCounts     <- 10
minConditions <- 4
DE_bwa_col5$counts <- DE_bwa_col5$counts[apply(DE_bwa_col5$counts >= minCounts, 1, sum) >= minConditions, ]

#separating host transcripts
hostNames   <- scan('../hostNames_ITpooled_20150526_cd-hit-est_0.90.txt', what='character')
hostCounts  <- DE_bwa_col5[rownames(DE_bwa_col5) %in% hostNames, ]

#running glm approach, accounting for two conditions in the model - pH and day of sampling
diff_pH <-
factor(c("ph7.6","ph7.6","ph7.6","ph7.6","ph7.9","ph7.9","ph7.9","ph7.9","ph8.2","ph8.2","ph8.2","ph8.2"))
day <- factor(c("13/5","14/5","13/5","14/5","13/5","14/5","13/5","14/5","13/5","14/5","13/5","14/5"))
hostCounts_df     <- data.frame(Sample=colnames(hostCounts),diff_pH,day)
design      <- model.matrix(~0 + diff_pH + day, data=hostCounts_df)
colnames(design)  <- c("pH7.6", "pH7.9", "pH8.2","day2")

#extracting only DE transcripts affected by pH condition variable (here between pH 7.9 and pH 8.2 conditions)
setwd("F:/results/")
contrastss <- makeContrasts(pH8.2 - pH7.9, levels = design)
y     <- estimateDisp(hostCounts, design)
fit   <- glmFit(y, design)
lrt   <- glmLRT(fit, contrast = contrastss)
is.de <- decideTestsDGE(lrt, p.value=0.05)
summary(is.de)
FDR   <- p.adjust(lrt$table$PValue, method="BH")
lrt_pH8.2vs7.9 <- topTags(lrt, n=nrow(lrt$table), sort.by = "PValue", p.value = 0.05)
write.csv(lrt_pH8.2vs7.9, file='DE transcripts in pH 8.2 vs pH 7.9 - glm contrasts.csv')
```

```
#extracting only DE transcripts affected by pH condition variable (here between pH 7.6 and pH 8.2 conditions)
contrastss2    <- makeContrasts(pH8.2 - pH7.6, levels = design)
y2    <- estimateDisp(hostCounts, design)
fit2  <- glmFit(y2, design)
lrt2  <- glmLRT(fit2, contrast = contrastss2)
is.de2 <- decideTestsDGE(lrt2, p.value=0.05)
summary(is.de2)
lrt_pH8.2vs7.6 <- topTags(lrt2, n=nrow(lrt2$table), sort.by = "PValue", p.value = 0.05)
write.csv(lrt_pH8.2vs7.6, file='DE transcripts in pH 8.2 vs pH 7.6 - glm contrasts.csv')

#extracting only DE transcripts affected by pH condition variable (here between pH 7.9 and pH 7.6 conditions)
contrastss3    <- makeContrasts(pH7.9 - pH7.6, levels = design)
y3    <- estimateDisp(hostCounts, design)
fit3  <- glmFit(y3, design)
lrt3  <- glmLRT(fit3, contrast = contrastss3)
is.de3 <- decideTestsDGE(lrt3, p.value=0.05)
summary(is.de3)
lrt_pH7.9vs7.6 <- topTags(lrt3, n=nrow(lrt3$table), sort.by = "PValue", p.value = 0.05)
write.csv(lrt_pH7.9vs7.6, file='DE transcripts in pH 7.9 vs pH 7.6 - glm contrasts.csv')

#merging the DE lists
list1 <- merge(lrt_pH8.2vs7.9, lrt_pH8.2vs7.6, all.x = T, all.y = T, by.x = "row.names", by.y = "row.names", na = T)
all_DEtranscripts <- merge(list1, lrt_pH7.9vs7.6, all.x = T, all.y = T, by.x = "Row.names", by.y = "row.names", na =
T)
unique(all_DEtranscripts$Row.names)

#pipeline for symbiont - inputting the count files, creating DGE file and performing filtering
setwd("F:/abundance_estimation_new/bwa_express")
files_bwa <- c("ph7.6_1.xprs","ph7.6_2.xprs","ph7.6_3.xprs","ph7.6_4.xprs",
               "ph7.9_1.xprs","ph7.9_2.xprs","ph7.9_3.xprs","ph7.9_4.xprs",
               "ph8.2_1.xprs","ph8.2_2.xprs","ph8.2_3.xprs","ph8.2_4.xprs")
group <- factor(c(1,1,1,1,2,2,2,2,3,3,3,3))
DE_bwa_col5 <- readDGE(files_bwa, path=NULL, columns=c(2, 5), group=group, labels=NULL)
minCounts     <- 10
minConditions <- 4
DE_bwa_col5$counts <- DE_bwa_col5$counts[apply(DE_bwa_col5$counts >= minCounts, 1, sum) >= minConditions, ]

#separating symbiont transcripts
symbNames   <- scan('../symbNames_ITpooled_20150526_cd-hit-est_0.90.txt', what='character')
symbCounts  <- DE_bwa_col5[rownames(DE_bwa_col5) %in% symbNames, ]
```

```
#running glm approach, accounting for two conditions in the model - pH and day of sampling
diff_pH <-
factor(c("ph7.6","ph7.6","ph7.6","ph7.6","ph7.9","ph7.9","ph7.9","ph7.9","ph8.2","ph8.2","ph8.2","ph8.2"))
day <- factor(c("13/5","14/5","13/5","14/5","13/5","14/5","13/5","14/5","13/5","14/5","13/5","14/5"))
symbCounts_df    <- data.frame(Sample=colnames(symbCounts),diff_pH,day)
design  <- model.matrix(~0 + diff_pH + day, data=symbCounts_df)
colnames(design) <- c("pH7.6", "pH7.9", "pH8.2","day2")

#extracting only DE transcripts affected by pH condition variable (here between pH 7.9 and pH 8.2 conditions)
setwd("F:/results/")
contrastss_s     <- makeContrasts(pH8.2 - pH7.9, levels = design)
y_s   <- estimateDisp(symbCounts, design)
fit_s <- glmFit(y_s, design)
lrt_s <- glmLRT(fit_s, contrast = contrastss_s)
is.de_s <- decideTestsDGE(lrt_s, p.value=0.05)
summary(is.de_s)
lrt_pH8.2vs7.9s  <- topTags(lrt_s, n=nrow(lrt_s$table), sort.by = "PValue", p.value = 0.05)
write.csv(lrt_pH8.2vs7.9s, file='DE transcripts in pH 8.2 vs pH 7.9 - symbiont - glm contrasts.csv')

#extracting only DE transcripts affected by pH condition variable (here between pH 7.6 and pH 8.2 conditions)
contrastss2_s    <- makeContrasts(pH8.2 - pH7.6, levels = design)
y2_s   <- estimateDisp(symbCounts, design)
fit2_s <- glmFit(y2_s, design)
lrt2_s <- glmLRT(fit2_s, contrast = contrastss2_s)
is.de2_s <- decideTestsDGE(lrt2_s, p.value=0.05)
summary(is.de2_s)
lrt_pH8.2vs7.6s <- topTags(lrt2_s, n=nrow(lrt2_s$table), sort.by = "PValue", p.value = 0.05)
write.csv(lrt_pH8.2vs7.6s, file='DE transcripts in pH 8.2 vs pH 7.6 - symbiont - glm contrasts.csv')

#extracting only DE transcripts affected by pH condition variable (here between pH 7.9 and pH 7.6 conditions)
contrastss3_s   <- makeContrasts(pH7.9 - pH7.6, levels = design)
y3_s   <- estimateDisp(symbCounts, design)
fit3_s <- glmFit(y3_s, design)
lrt3_s <- glmLRT(fit3_s, contrast = contrastss3_s)
is.de3_s <- decideTestsDGE(lrt3_s, p.value=0.05)
summary(is.de3_s)
lrt_pH7.9vs7.6s <- topTags(lrt3_s, n=nrow(lrt3_s$table), sort.by = "PValue", p.value = 0.05)
write.csv(lrt_pH7.9vs7.6s, file='DE transcripts in pH 7.9 vs pH 7.6 - symbiont - glm contrasts.csv')
```

```
#merging the DE lists
list1s <- merge(lrt_pH8.2vs7.9s, lrt_pH8.2vs7.6s, all.x = T, all.y = T, by.x = "row.names", by.y = "row.names", na = 
T)
all_DEtranscripts_s <- merge(list1s, lrt_pH7.9vs7.6s, all.x = T, all.y = T, by.x = "Row.names", by.y = "row.names", 
na = T)
unique(all_DEtranscripts_s$Row.names)
```
