# Supplementary material for: Ocean acidification at a coastal CO2 vent induces expression of stress-related transcripts and transposable elements in the sea anemone Anemonia viridis
Source: PLoS One. 2019 May 8;14(5):e0210358. doi: 10.1371/journal.pone.0210358 (PMC6505742; doi:10.1371/journal.pone.0210358)
Supplement: S5 Table — Shown is the R script used to find enriched gene ontology (GO) categories at pH 7.6 compared to normal seawater pH 8.2. (PDF) [file pone.0210358.s008.pdf]

S5 Table. R script for gene set enrichment analyses.

```
library(goseq)
sizes         <- read.table(file="F:/goseq/Avir_ITpooled_NewlyExported.txt", Sep="\t", header=T)
assayed.genes <- sizes$genes
de.genes      <- rownames(expVal1)
gene.vector    = as.integer(assayed.genes%in%de.genes)
names(gene.vector)   = assayed.genes
geneSize      <- sizes$seq_length
names(geneSize)     <- sizes$genes
pwf=nullp(gene.vector, bias.data=geneSize)
GOs           <- read.delim(file='F:/new_goseq/annot_Avir.txt', sep='\t', header=F)
colnames(GOs) <- c("genes", "GO_term","GO_number","general_term","GO_process")
category_mappings   <- GOs[c(1,3)]
GO.wall        = goseq(pwf,gene2cat=category_mappings,use_genes_without_cat=TRUE)
enriched.GO    = GO.wall$category[p.adjust(GO.wall$over_represented_pvalue,method="BH")<.05]
library(GO.db)
for(go in enriched.GO){
  print(GOTERM[[go]])
  cat("--------------------------------------\n")
}

GO.enriched_pvalue  <- GO.wall[GO.wall$category %in% enriched.GO, ]
GO.enriched_withNames <- merge (GO.enriched_pvalue, category_mappings, all.x = T, all.y = F, by.x = "row.names",
by.y = "row.names", na = T)
write.csv(GO.enriched_withNames, file='GO.enriched_withNames.csv')
```
