# Supplementary material for: Ocean acidification at a coastal CO2 vent induces expression of stress-related transcripts and transposable elements in the sea anemone Anemonia viridis
Source: PLoS One. 2019 May 8;14(5):e0210358. doi: 10.1371/journal.pone.0210358 (PMC6505742; doi:10.1371/journal.pone.0210358)
Supplement: S6 Table — A list of up- and down-regulated gene ontology (GO) categories in Anemonia viridis detected at low pH 7.6 compared to normal seawater pH 8.2. (PDF) [file pone.0210358.s009.pdf]

S6 Table. Enriched gene ontology categories at pH 7.6.

A) Up-regulated GO categories

GO term [1]	GO category [2]	p-value [3]	NumDEInCat [4]	NumInCat [5]	Ontology [6]	% DE [7]
**endoplasmic reticulum lumen**	GO:0005788	4.60E-08	6	63	CC	9.5%
**heme oxygenase (decyclizing) activity**	GO:0004392	6.71E-08	3	5	MF	60.0%
**heme oxidation**	GO:0006788	6.71E-08	3	5	BP	60.0%
**endocytic vesicle lumen**	GO:0071682	4.72E-06	2	2	CC	100%
**endoplasmic reticulum chaperone complex**	GO:0034663	1.43E-05	3	15	CC	20.0%
**unfolded protein binding**	GO:0051082	2.22E-05	6	200	MF	3.0%

[1]Gene ontology (GO) term.

[2]Gene ontology (GO) category.

[3]Significant p-values are shown. GO categories were considered as enriched only if FDR-adjusted p-value < 0.05.

[4]Number of differentially expressed transcripts in the GO category.

[5]Number of transcripts in the GO category present in the dataset.

[6] Gene ontology domain (BP – biological process, CC – cellular component and MF – molecular function).

[7]Percent of differentially expressed transcripts in the GO category.

B) Down-regulated GO categories

GO term [1]	GO category [2]	Over-represented p-value [3]	NumDEInCat [4]	NumInCat [5]	Ontology [6]	% DE [7]
**zymogen binding**	GO:0035375	2.98E-07	3	7	MF	42.9%
**zymogen granule membrane**	GO:0042589	1.39E-06	3	11	CC	27.3%
**positive regulation of epithelial cell differentiation**	GO:0030858	1.73E-06	3	11	BP	27.3%
**inner cell mass cell proliferation**	GO:0001833	6.12E-06	3	17	BP	17.7%
**NAD+ synthase activity**	GO:0008795	1.47E-05	2	3	MF	66.7%
**extracellular matrix**	GO:0031012	1.80E-05	4	72	CC	5.6%
**phagocytic vesicle membrane**	GO:0030670	1.81E-05	3	24	CC	12.5%

[1]Gene ontology (GO) term.
[2]Gene ontology (GO) category.
[3]Significant p-values are shown. GO categories were considered as enriched only if FDR-adjusted p-value < 0.05.
[4]Number of differentially expressed transcripts in the GO category.
[5]Number of transcripts in the GO category present in the dataset.
[6] Gene ontology domain (BP – biological process, CC – cellular component and MF – molecular function).
[7]Percent of differentially expressed transcripts in the GO category.
