# Supplementary material for: Ocean acidification at a coastal CO2 vent induces expression of stress-related transcripts and transposable elements in the sea anemone Anemonia viridis
Source: PLoS One. 2019 May 8;14(5):e0210358. doi: 10.1371/journal.pone.0210358 (PMC6505742; doi:10.1371/journal.pone.0210358)
Supplement: S7 Table — Shown is an extended list of significantly differentially expressed transcripts at low seawater pH 7.6 compared to normal seawater pH 8.2 in A. viridis from glm edgeR analysis (FDR < 0.05). (PDF) [file pone.0210358.s010.pdf]

S7 Table. Selected differentially expressed transcripts at low pH compared to normal seawater pH in *A. viridis*.

A) Heat shock proteins, molecular chaperones and additional stress-response genes

**Transcripts** [1]	**Transcript**	**LogFC** [2]	**p-value** [3]	**FDR** [4]	**Length** [5]	**e-value** [6]	**Blast similarity [%]** [7]
**heat shock protein 70**	TR56459|c7_g6_i1	7.80	9.50E-09	2.49E-05	399	7.42E-11	76.95
**heat shock 70 kda protein 1-like**	TR56459|c7_g9_i1	7.19	3.72E-09	1.24E-05	1900	0	89.05
**heat shock protein 70 partial**	TR9490|c3_g1_i1	6.05	3.06E-08	5.62E-05	374	2.36E-36	97.20
**heat shock 70 kda protein cognate 3**	TR38548|c0_g1_i1	3.14	2.67E-11	2.85E-07	392	9.60E-57	85.25
**heme oxygenase 1**	TR9534|c0_g1_i1	3.07	3.42E-05	8.62E-03	401	3.00E-08	65.91
**heat shock protein HSP 90-alpha-like**	TR59978|c0_g1_i1	3.06	1.12E-09	4.27E-06	294	9.00E-06	92.59
**heat shock protein 90**	TR15677|c3_g2_i1	2.76	4.22E-06	2.13E-03	2783	0	90.40
**heat shock protein 105 kda isoform x1**	TR56430|c0_g3_i1	2.69	5.61E-07	5.04E-04	1171	5.88E-159	74.40
**90-kDa heat-shock protein, partial**	TR61141|c0_g1_i1	2.56	1.87E-05	5.52E-03	402	6.00E-08	96.67
**heat shock protein 90kda alpha class a member gene 2**	TR63975|c0_g2_i1	2.47	8.68E-05	1.54E-02	237	1.79E-09	75.00
**hsp70 chaperone**	TR30554|c0_g1_i1	2.36	1.50E-05	4.82E-03	542	3.73E-11	62.95
**nuclear factor erythroid 2-related factor 1 isoform x3**	TR506|c0_g1_i1	2.35	2.24E-06	1.36E-03	2220	1.42E-31	59.90
**protein disulfide-isomerase a4**	TR71845|c2_g5_i3	2.27	3.78E-06	1.96E-03	2625	0	71.65
**glucose-regulated protein 78**	TR44825|c0_g1_i1	2.23	1.75E-06	1.14E-03	364	4.96E-15	72.30
**cyclic AMP-responsive element-binding protein 3-like protein 3-A**	TR67696|c1_g12_i1	2.23	1.71E-05	5.27E-03	304	2.00E-10	48.04
**78 kda glucose-regulated protein**	TR8826|c2_g2_i1	2.21	5.31E-06	2.49E-03	2503	0	93.00
**heat shock protein 90**	TR62660|c1_g1_i1	2.13	1.34E-04	2.00E-02	346	6.36E-06	78.25
**heat shock 70 kda protein 4l**	TR69486|c2_g4_i3	2.12	6.69E-05	1.29E-02	2049	5.94E-92	62.40
**90-kDa heat-shock protein, partial**	TR22696|c2_g4_i1	2.07	1.74E-04	2.33E-02	427	2.00E-08	96.77
**heat shock 70 kda protein 4l**	TR69486|c2_g4_i5	2.01	1.60E-04	2.23E-02	1992	2.00E-79	60.25

**hypoxia up-regulated protein 1-like**	TR82131|c1_g7_i3	1.95	7.81E-05	1.43E-02	2474	0	76.35
**hypoxia up-regulated protein 1**	TR82131|c1_g9_i1	1.78	4.43E-04	4.28E-02	357	2.50E-24	64.90
**hypoxia up-regulated protein 1 isoform x1**	TR82131|c1_g8_i1	1.77	2.88E-04	3.27E-02	761	5.42E-79	65.25
**heat shock protein 70**	TR29900|c0_g1_i4	1.71	3.65E-04	3.84E-02	364	1.28E-07	76.67
**heat shock protein 83**	TR41150|c2_g1_i1	1.61	2.54E-04	3.00E-02	1969	5.77E-75	59.80
**glucose-regulated protein 94**	TR41995|c0_g2_i1	1.60	5.61E-04	4.96E-02	2534	0	85.80
**peroxiredoxin-4 isoform x2**	TR11067|c1_g5_i1	-2.81	2.60E-04	3.04E-02	245	9.18E-19	91.85
**thioredoxin domain-containing protein 11**	TR10921|c0_g1_i1	-4.12	2.21E-04	2.77E-02	889	5.14E-17	44.11
**heat shock protein 70**	TR56459|c7_g6_i1	7.80	9.50E-09	2.49E-05	399	7.42E-11	76.95

B) Metabolism

**Transcripts [1]**	**Transcript**	**LogFC [2]**	**p-value [3]**	**FDR [4]**	**Length [5]**	**e-value [6]**	**Blast similarity [%] [7]**
**mitochondrial enolase superfamily member 1-like**	TR52271|c2_g3_i4	3.46	2.25E-04	2.81E-02	1668	0.00E+00	81.05
**mitochondrial enolase superfamily member 1**	TR52271|c2_g3_i3	3.34	4.31E-04	4.21E-02	1762	0	79.30
**fad linked oxidase domain-containing protein**	TR3752|c1_g1_i1	2.65	9.15E-06	3.59E-03	2361	1.40E-50	45.85
**n-acetyltransferase 9**	TR55638|c4_g1_i1	2.46	1.69E-04	2.29E-02	294	5.16E-28	82.50
**thioredoxin-like protein 4b**	TR72319|c0_g2_i2	2.45	1.81E-05	5.38E-03	313	7.55E-23	84.65
**succinate--CoA ligase [ADP-forming] subunit beta, mitochondrial-like**	TR19057|c0_g1_i3	2.32	3.57E-04	3.78E-02	446	1.00E-08	96.55
**piRNA biogenesis protein EXD1-like**	TR26746|c0_g2_i1	2.30	1.83E-04	2.43E-02	418	2.00E-15	62.86
**probable trna (guanine -n)-dimethyltransferase-like**	TR11050|c1_g1_i1	2.12	5.67E-04	4.98E-02	785	4.77E-28	70.95
**isocitrate lyase**	TR53810|c0_g1_i1	2.03	3.95E-05	9.44E-03	2410	0.00E+00	81.50
**3-methyl-2-oxobutanoate dehydrogenase**	TR79493|c0_g2_i3	1.98	1.24E-04	1.93E-02	988	7.62E-95	72.75
**heme oxygenase 2-like**	TR2095|c0_g3_i1	1.97	9.77E-05	1.67E-02	299	2.90E-43	75.20
**heme oxygenase 2-like**	TR2095|c0_g9_i1	1.93	1.18E-04	1.87E-02	392	1.28E-64	77.65
**cytochrome p450 3a40-like**	TR18712|c0_g2_i1	1.88	5.54E-04	4.93E-02	1415	4.71E-21	57.45
**stomatin-like protein 2**	TR34115|c2_g7_i1	1.81	5.44E-04	4.88E-02	344	2.28E-35	77.70

**heme oxygenase**	TR2095|c0_g8_i1	1.70	2.63E-04	3.05E-02	1012	1.44E-47	67.10
**spermidine synthase**	TR51582|c0_g1_i1	-1.50	3.25E-04	3.55E-02	228	1.47E-17	83.05
**acyl-lipid (8-3)-desaturase-like**	TR26197|c0_g1_i1	-1.74	2.68E-04	3.10E-02	470	6.00E-09	92.59
**niemann-pick c 2 like**	TR4599|c1_g6_i3	-1.78	8.88E-05	1.56E-02	818	4.97E-29	55.20
**adp-ribosyl cyclase precursor**	TR41278|c0_g1_i1	-1.92	1.85E-05	5.50E-03	963	1.87E-36	52.75
**amidohydrolase family protein**	TR52385|c0_g2_i1	-2.08	1.67E-04	2.27E-02	365	1.32E-16	74.30
**phospholipase a2**	TR671|c0_g3_i1	-2.31	5.69E-04	4.99E-02	807	6.50E-27	57.00
**galactoside 3(4)-L-fucosyltransferase-like**	TR34495|c1_g2_i1	-2.43	1.12E-05	4.04E-03	232	1.00E-06	55.56
**glutamine-dependent nad(+) synthetase**	TR21274|c4_g13_i1	-2.52	3.57E-04	3.78E-02	552	3.96E-96	84.90
**cytochrome oxidase subunit 1, partial**	TR69534|c2_g8_i1	-2.56	5.66E-07	5.04E-04	276	3.31E-39	99.05
**protein-glutamine gamma-glutamyltransferase k-like**	TR55703|c5_g2_i1	-2.96	1.90E-04	2.48E-02	1288	1.25E-169	67.80
**glutamine-dependent nad(+) synthetase**	TR21274|c4_g9_i2	-3.13	5.50E-06	2.51E-03	2752	0	78.70
**beta-carotenoid oxygenase**	TR73138|c1_g1_i4	-3.20	5.93E-05	1.22E-02	229	4.21E-12	77.75
**rna pseudouridylate synthase domain-containing protein 4-like**	TR39929|c1_g4_i1	-3.30	1.13E-05	4.04E-03	612	8.64E-21	72.50

C) Cell growth and survival genes

Transcripts [1]	Transcript	LogFC [2]	p-value [3]	FDR [4]	Length [5]	e-value [6]	Blast similarity [%] [7]
**neogenin isoform x2**	TR30053|c1_g1_i3	3.65	3.92E-04	4.04E-02	1028	1.60E-08	53.70
**probable e3 ubiquitin-protein ligase ari9- partial**	TR34665|c0_g2_i1	3.29	5.02E-06	2.39E-03	710	1.34E-53	64.15
**ornithine decarboxylase**	TR2341|c0_g1_i1	2.83	1.37E-07	1.92E-04	561	1.98E-34	62.55
**Bcl-2-like 2 protein (BCL2L2)**	TR18835|c6_g1_i1	2.56	5.28E-05	1.12E-02	1132	7,01E-58	78.00
**eukaryotic translation initiation factor 2-alpha kinase 3-like (EIF2AK3)**	TR5221|c2_g2_i1	2.56	1.14E-05	4.04E-03	269	2.00E-07	59.52
**eukaryotic translation initiation factor 2-alpha kinase 3 (EIF2AK3)**	TR5221|c2_g3_i2	2.53	1.56E-05	4.94E-03	2885	0.00E+00	52.65
**nuclear factor nf-kappa-b p100 subunit**	TR20187|c0_g1_i1	2.22	2.49E-04	2.96E-02	2584	1.02E-71	56.55
**probable e3 ubiquitin-protein ligase dtx3**	TR72192|c0_g1_i1	2.04	1.62E-04	2.25E-02	757	2.52E-47	72.65

**probable e3 ubiquitin-protein ligase dtx3**	TR72192|c0_g1_i3	1.96	9.39E-05	1.62E-02	1654	2.99E-43	69.75
**serine threonine-protein kinase pats1**	TR3735|c2_g1_i1	-1.70	1.34E-04	2.00E-02	2089	7.21E-155	51.95
**fibroblast growth factor receptor 2-like**	TR69774|c0_g4_i2	-2.94	5.78E-05	1.19E-02	1023	1.00E-06	37.65
**haus augmin-like complex subunit 3**	TR22306|c0_g2_i1	-3.58	7.72E-06	3.22E-03	376	6.24E-10	70.00
**dna repair protein rev1-like**	TR91710|c0_g1_i1	-4.10	7.89E-06	3.24E-03	399	5.99E-24	66.45
**matrix metalloproteinase-9**	TR30019|c0_g3_i5	-4.14	1.05E-06	7.68E-04	913	1.18E-26	57.60

D) Signal transduction pathways and membrane trafficking genes

**Transcripts [1]**	**Transcript**	**LogFC [2]**	**p-value [3]**	**FDR [4]**	**Length [5]**	**e-value [6]**	**Blast similarity [%] [7]**
**rho-related btb domain-containing protein 3**	TR25831|c1_g1_i3	3.91	7.08E-11	6.29E-07	1198	1.59E-48	53.8
**receptor-type tyrosine-protein phosphatase delta**	TR54344|c3_g6_i2	3.08	2.62E-05	7.25E-03	1013	1.00E-42	35.84
**hedgehog interacting protein**	TR68586|c0_g1_i1	2.40	9.62E-06	3.75E-03	362	4.40E-09	53.91
**regulating synaptic membrane exocytosis protein 2-like**	TR29084|c0_g1_i1	2.33	2.93E-06	1.61E-03	259	4.34E-15	77.75
**synaptosomal-associated protein 29-like (SNAP29)**	TR86900|c0_g1_i1	2.15	1.17E-04	1.85E-02	1210	1.58E-63	50.5

E) Immune and antiviral response genes

**Transcripts [1]**	**Transcript**	**LogFC [2]**	**p-value [3]**	**FDR [4]**	**Length [5]**	**e-value [6]**	**Blast similarity [%] [7]**
**interferon regulatory factor 2**	TR152|c1_g1_i1	2.58	2.09E-05	6.07E-03	1320	7.12E-35	69.70
**interferon-induced helicase c domain-containing protein 1**	TR72169|c1_g1_i2	2.10	2.35E-04	2.88E-02	2470	0.00E+00	55.10
**complement factor b precursor**	TR1731|c0_g1_i2	-1.45	3.58E-04	3.78E-02	2443	0.00E+00	50.25
**complement factor b precursor**	TR1731|c0_g1_i1	-1.51	2.46E-04	2.93E-02	2377	0.00E+00	52.10
**scavenger receptor cysteine-rich domain-containing**	TR72724|c0_g1_i1	-2.38	1.04E-04	1.72E-02	321	1.82E-06	59.33

group b							
**coagulation factor ix**	TR32255|c1_g1_i1	-2.44	8.04E-06	3.25E-03	1163	7.88E-68	60.10
**probable chitinase 3-like**	TR69465|c4_g13_i1	-2.74	6.12E-05	1.22E-02	994	1.37E-07	60.00
**deleted in malignant brain tumors 1**	TR21259|c7_g3_i1	-5.95	1.85E-10	1.23E-06	652	4.71E-61	65.6

F) Transport channels

Transcripts [1]	Transcript	LogFC [2]	p-value [3]	FDR [4]	Length [5]	e-value [6]	Blast similarity [%] [7]
**sodium hydrogen exchanger 10-like**	TR2677|c1_g3_i2	3.63	4.73E-05	1.05E-02	2906	0	56.65
**sodium hydrogen exchanger 10-like**	TR2677|c1_g3_i4	3.47	1.94E-04	2.52E-02	2942	0	53.55
**voltage-gated sodium channel**	TR101422|c0_g1_i1	2.06	1.02E-04	1.70E-02	579	2.15E-50	71.55
**voltage-gated sodium channel**	TR63268|c0_g2_i1	-1.62	1.22E-04	1.90E-02	401	4.56E-39	62.15

G) Others

Transcripts [1]	Transcript	LogFC [2]	p-value [3]	FDR [4]	Length [5]	e-value [6]	Blast similarity [%] [7]
**putative skeletal organic matrix protein 3**	TR52199|c0_g3_i2	-2.07	1.02E-04	1.71E-02	961	1.00E-27	29.06
**Ate-SCRiP Small cysteine-rich protein**	TR5921|c3_g2_i1	-2.36	4.44E-04	4.29E-02	803	4.00E-08	44.83
**putative skeletal organic matrix protein 3**	TR52199|c0_g5_i1	-2.41	1.52E-06	1.01E-03	594	2.00E-16	40.24

[1] Presented is a list of selected differentially expressed genes at pH 7.6 compared to pH 8.2 (normal conditions) in Anemonia viridis from six different categories:
A) Heat shock proteins, molecular chaperones and additional stress-response genes,
B) Metabolism,
C) Cell growth and survival genes,
D) Signal transduction pathways and membrane trafficking genes,
E) Immune and antiviral response genes
F) Transport channels, and
G) Others.

[2] *Shown are logarithmic fold change values (log_2FC) as output from edgeR analysis. Both upregulated transcripts (positive values) and downregulated transcripts (negative values) at pH 7.6 compared to normal conditions are presented in the same column.*

[3,4] *p-values and False Discovery Rate (FDR) values from edgeR analysis are shown. Transcripts were considered as differentially expressed only if FDR-adjusted p-value < 0.05.*

[5] *Transcript length.*

[6,7] *e-value and blast similarity values are shown to support confidence in names assigned to our transcripts.*
