# Supplementary material for: Ocean acidification at a coastal CO2 vent induces expression of stress-related transcripts and transposable elements in the sea anemone Anemonia viridis
Source: PLoS One. 2019 May 8;14(5):e0210358. doi: 10.1371/journal.pone.0210358 (PMC6505742; doi:10.1371/journal.pone.0210358)
Supplement: S8 Table — Shown is a list of selected differentially expressed transcripts at (A) pH 7.6 and (B) pH 7.9 compared to normal seawater pH 8.2 in Symbiodinium sp. from glm edgeR analysis (FDR < 0.05). (PDF) [file pone.0210358.s011.pdf]

S8 Table. Selected differentially expressed transcripts at low pH compared to normal seawater pH in *Symbiodinium* sp.

1) pH 7.6

A) Heat shock proteins, molecular chaperones and additional stress-response genes

Transcripts [1]	Transcript	LogFC [2]	p-value [3]	FDR [4]	Length [5]	e-value [6]	Blast similarity [%] [7]
**heat shock protein 83**	TR7328|c0_g1_i1	2.29	1.27E-04	3.86E-02	764	1.15E-12	97.00
**stress-induced-phosphoprotein 1**	TR77930|c0_g1_i1	-2.55	8.42E-04	7.06E-02	989	1.45E-52	63.90

B) Metabolism

Transcripts [1]	Transcript	LogFC [2]	p-value [3]	FDR [4]	Length [5]	e-value [6]	Blast similarity [%] [7]
**peroxisomal bifunctional enzyme**	TR49448|c0_g2_i1	2.27	1.32E-06	2.13E-03	425	4.00E-20	64.86
**phosphatidate phosphatase app1**	TR2711|c0_g1_i1	-1.64	4.46E-05	1.91E-02	2211	1.16E-36	55.3
**ATP-citrate synthase**	TR5991|c0_g1_i1	-3.51	3.12E-05	1.53E-02	432	1.00E-13	54.37
**glutamine synthetase**	TR25876|c0_g5_i1	-3.67	3.24E-05	1.56E-02	283	5.59E-13	59.38
**dimethylglycine mitochondrial**	TR78065|c0_g1_i1	-4.05	1.22E-04	1.95E-02	268	1.21E-13	69.5
**bifunctional arginine demethylase and lysyl-hydroxylase JMJD6-A**	TR71082|c0_g1_i1	-4.92	1.11E-06	1.93E-03	340	4.00E-28	80.95

C) Signal transduction pathways, cell growth and membrane trafficking genes

Transcripts [1]	Transcript	LogFC [2]	p-value [3]	FDR [4]	Length [5]	e-value [6]	Blast similarity [%] [7]
**putative metabolite transport protein GIT1**	TR5296|c1_g8_i1	4.00	1.25E-04	3.86E-02	264	2.00E-13	64.81
**synaptic vesicle 2-related protein**	TR36565|c0_g1_i1	3.91	2.48E-05	1.47E-02	837	1.00E-10	47.90
**protein serine threonine kinase**	TR21314|c0_g1_i1	2.61	9.76E-05	1.74E-02	725	1.98E-13	49.50
**calcium-dependent protein kinase 1**	TR88949|c0_g1_i1	2.52	5.89E-06	5.56E-03	317	4.00E-23	85.45
**autophagy 8i**	TR66102|c0_g1_i1	2.43	4.23E-05	1.90E-02	242	2.26E-07	64.00
**methionine aminopeptidase 2B**	TR101509|c0_g1_i1	-1.66	1.61E-04	4.45E-02	258	1.00E-25	81.03

**calcium-dependent protein kinase 2**	TR12292|c0_g1_i1	-3.29	1.10E-04	3.62E-02	402	2.00E-28	68.81
**D-xylose-proton symporter-like 1**	TR59696|c0_g1_i1	-3.43	2.13E-05	1.34E-02	428	2.00E-08	71.05
**calmodulin-4**	TR14432|c0_g1_i1	-3.60	2.99E-05	1.52E-02	1576	3.00E-157	56.54
**eukaryotic translation initiation factor isoform 4G-2**	TR1391|c0_g1_i1	-3.76	2.20E-06	2.93E-03	800	1.00E-98	90.83
**ADP-ribosylation factor 1**	TR28038|c0_g1_i1	-3.78	3.35E-05	1.58E-02	307	2.00E-13	84.62
**cyclin-dependent kinase 10**	TR45223|c0_g1_i1	-4.83	1.07E-07	4.05E-04	276	2.00E-27	76.56
**protein-lysine methyltransferase METTL21B**	TR92153|c0_g1_i1	-4.96	6.98E-06	5.86E-03	286	2.00E-12	51.61
**mitogen-activated protein kinase kinase kinase 2**	TR49882|c0_g1_i1	-5.65	2.05E-07	5.81E-04	353	7.00E-52	90.43

D) Transport channels

**Transcripts [1]**	**Transcript**	**LogFC [2]**	**p-value [3]**	**FDR [4]**	**Length [5]**	**e-value [6]**	**Blast similarity [%] [7]**
**voltage-dependent N-type calcium channel subunit alpha-1B**	TR53155|c0_g1_i1	2.31	1.20E-04	3.79E-02	336	3.00E-34	92.96
**voltage-gated ion channel superfamily**	TR73007|c0_g1_i1	2.24	2.53E-05	1.47E-02	584	1.66E-10	48.85
**potassium voltage-gated channel subfamily H member 1**	TR93867|c0_g1_i1	-2.57	6.69E-06	5.83E-03	288	7.00E-12	48.33
**ion channel castor-like**	TR25593|c0_g1_i1	-3.27	2.24E-04	2.81E-02	1589	1.95E-20	46.00

[1] *Presented is a list of selected differentially expressed genes at pH 7.6 compared to pH 8.2 (normal conditions) in Anemonia viridis from six different categories:*

A) Heat shock proteins, molecular chaperones and additional stress-response genes,

B) Metabolism,

C) Signal transduction pathways, cell growth and membrane trafficking genes, and

D) Transport channels.

[2] *Shown are logarithmic fold change values (logFC or log_2FC) as output from edgeR analysis. Both upregulated transcripts (positive values) and downregulated transcripts (negative values) at pH 7.6 compared to normal conditions are presented in the same column.*

[3,4] *p-values and False Discovery Rate (FDR) values from edgeR analysis are shown. Transcripts were considered as differentially expressed only if FDR-adjusted p-value < 0.05.*

[5] *Transcript length.*

[6,7] *e-value and blast similarity values are shown to support confidence in names assigned to our transcripts.*

2) pH 7.9

A) Heat shock proteins, molecular chaperones and additional stress-response genes

Transcripts [1]	Transcript	LogFC [2]	p-value [3]	FDR [4]	Length [5]	e-value [6]	Blast similarity [%] [7]
**molecular chaperone**	TR60320|c14_g1_i1	2.74	5.87E-06	3.60E-03	518	1.87E-17	54.37
**stress-induced-phosphoprotein 1**	TR77930|c0_g1_i1	-2.38	4.59E-04	4.05E-02	989	1.45E-52	63.90

B) Metabolism

Transcripts [1]	Transcript	LogFC [2]	p-value [3]	FDR [4]	Length [5]	e-value [6]	Blast similarity [%] [7]
**Mitochondrial intermediate peptidase**	TR31504|c0_g2_i1	2.40	2.59E-04	4.42E-02	731	5.00E-24	54.79
**Succinate dehydrogenase assembly factor 2, mitochondrial**	TR60308|c0_g2_i1	1.92	3.13E-05	1.15E-02	536	1.00E-30	83.96
**catalase peroxidase hpi**	TR73466|c0_g1_i1	-2.46	8.81E-07	2.23E-04	738	3.27E-38	60.45
**thioredoxin-like protein 4a**	TR13626|c0_g1_i1	-2.62	3.71E-05	1.25E-02	343	9.36E-21	58.25
**ubiquinone menaquinone biosynthesis methyltransferase**	TR104281|c0_g1_i1	-2.63	4.21E-05	1.29E-02	396	4.20E-20	59.15
**phosphoglucomutase**	TR22959|c0_g1_i1	-2.66	5.42E-08	2.46E-04	429	3.55E-40	70.75
**ATP-citrate synthase**	TR5991|c0_g1_i1	-3.00	2.57E-04	4.42E-02	432	1.00E-13	54.37
**glutamine synthetase**	TR25876|c0_g5_i1	-3.26	4.27E-06	8.71E-04	283	5.59E-13	59.38
**Peptidyl-tRNA hydrolase**	TR31589|c0_g1_i1	-3.54	4.30E-05	1.29E-02	282	2.00E-15	77.27
**dimethylglycine mitochondrial**	TR78065|c0_g1_i1	-3.62	5.93E-05	7.93E-03	268	1.21E-13	69.50
**Carboxylic acid reductase**	TR62853|c1_g1_i1	-3.79	1.45E-05	6.82E-03	233	2.00E-09	55.56
**type I polyketide synthase**	TR106174|c0_g1_i1	-4.18	1.89E-04	3.63E-02	422	4.00E-13	35.48
**Bifunctional arginine demethylase and lysyl-hydroxylase JMJD6-A**	TR71082|c0_g1_i1	-4.31	1.11E-06	1.93E-03	340	4.00E-28	80.95
**peptide methionine sulfoxide reductase**	TR99406|c0_g1_i1	-4.88	1.36E-07	4.40E-04	278	9.07E-11	68.80
**PI-PLC X domain-containing protein 1**	TR76563|c0_g1_i1	-4.91	1.32E-04	2.93E-02	459	3.00E-07	40.45

C) Signal transduction pathways, cell growth and membrane trafficking genes

Transcripts [1]	Transcript	LogFC [2]	p-value [3]	FDR [4]	Length [5]	e-value [6]	Blast similarity [%] [7]
**Methionine aminopeptidase 2B**	TR72258|c0_g1_i1	3.43	2.06E-04	3.80E-02	310	3.00E-36	82.14
**protein serine threonine kinase**	TR21314|c0_g1_i1	2.41	2.12E-04	2.21E-02	725	1.98E-13	49.50
**D-xylose-proton symporter-like 1**	TR59696|c0_g1_i1	-2.86	2.13E-05	1.34E-02	428	2.00E-08	71.05
**calcium-dependent protein kinase 1**	TR1194|c0_g1_i1	-3.07	4.87E-04	4.21E-02	1106	1.36E-09	64.15
**Tankyrase-2**	TR25917|c0_g2_i1	-3.23	2.97E-04	4.74E-02	268	2.00E-16	69.23
**Calcium-dependent protein kinase 26**	TR45376|c0_g2_i1	-3.42	6.67E-06	3.88E-03	783	2.00E-12	31.05
**Eukaryotic translation initiation factor isoform 4G-2**	TR1391|c0_g1_i1	-3.84	2.20E-06	2.93E-03	800	1.00E-98	90.83
**Calmodulin-4**	TR14432|c0_g1_i1	-3.94	6.14E-06	3.66E-03	1576	3.00E-157	56.54
**ADP-ribosylation factor 1**	TR28038|c0_g1_i1	-4.11	7.80E-06	4.26E-03	307	2.00E-13	84.62
**serine threonine-protein kinase nek1-like isoform x1**	TR19038|c0_g1_i1	-4.23	1.93E-04	3.68E-02	503	4.23E-13	50.70
**adenylate cyclase 1**	TR26368|c0_g4_i1	-4.62	7.31E-07	9.64E-04	1373	5.00E-58	57.20
**Tubby protein-like 1**	TR59667|c0_g1_i1	-6.31	1.39E-11	3.15E-07	295	3.00E-12	65.85

D) Transport channels

Transcripts [1]	Transcript	LogFC [2]	p-value [3]	FDR [4]	Length [5]	e-value [6]	Blast similarity [%] [7]
**Voltage-dependent calcium channel type A subunit alpha-1**	TR41083|c0_g1_i1	3.06	2.55E-05	9.98E-03	405	4.00E-40	69.91
**ion channel castor-like**	TR25593|c0_g1_i1	-2.90	1.79E-04	1.93E-02	1589	1.95E-20	46.00
**Sodium channel protein type 11 subunit alpha**	TR23787|c0_g1_i1	-4.47	2.82E-07	7.87E-04	565	7.00E-12	44.33
**Potassium voltage-gated channel subfamily A member 1**	TR82064|c1_g2_i1	-6.51	5.45E-07	9.49E-04	365	3.00E-21	84.48

E) Others

Transcripts [1]	Transcript	LogFC [2]	p-value [3]	FDR [4]	Length [5]	e-value [6]	Blast similarity [%] [7]
Carbonic anhydrase 2	TR85491|c0_g1_i1	-1.50	2.02E-04	3.77E-02	404	3.00E-10	62.22
Carbonic anhydrase 2	TR85491|c0_g2_i1	-1.54	1.41E-04	3.03E-02	595	4.00E-13	63.46

[1] *Presented is a list of selected differentially expressed genes at pH 7.9 compared to pH 8.2 (normal conditions) in Anemonia viridis from six different categories:*

A) Heat shock proteins, molecular chaperones and additional stress-response genes,

B) Metabolism,

C) Signal transduction pathways, cell growth and membrane trafficking genes,

D) Transport channels, and

E) Others.

[2] *Shown are logarithmic fold change values (logFC or log_2FC). Both upregulated transcripts (positive values) and downregulated transcripts (negative values) at pH 7.9 compared to normal conditions are presented in the same column.*

[3,4] *p-values and False Discovery Rate (FDR) values from edgeR analysis are shown. Transcripts were considered as differentially expressed only if FDR-adjusted p-value < 0.05.*

[5] *Transcript length.*

[6,7] *e-value and blast similarity values are shown to support confidence in names assigned to our transcripts.*
