# Supplementary material for: Ocean acidification at a coastal CO2 vent induces expression of stress-related transcripts and transposable elements in the sea anemone Anemonia viridis
Source: PLoS One. 2019 May 8;14(5):e0210358. doi: 10.1371/journal.pone.0210358 (PMC6505742; doi:10.1371/journal.pone.0210358)
Supplement: S9 Table — Protein-coding regions within differentially expressed transcripts were searched for domains or other functional signatures using InterPro database. Depicted is a list of recognized signatures that appeared up- or down-regulation in the symbiont at low seawater pH 7.6 compared to normal seawater pH 8.2. (PDF) [file pone.0210358.s012.pdf]

S9 Table. Analysis of protein signatures in the symbiont DE-data set.

Up-regulated InterPro signatures	Down-regulated InterPro signatures
Nitrogen permease regulator 2	Chlorophyll a/b binding domain superfamily
Palmitoyltransferase, DHHC domain	Chlorophyll A-B binding protein
Protein kinase domain	Cyclin
Protein kinase, ATP binding site	Cyclin D
Protein kinase-like domain superfamily	Cyclin-like superfamily
Protein kinase-like domain superfamily	DNA-directed RNA polymerase, subunit 2
Serine/threonine-protein kinase, active site	DNA-directed RNA polymerase, subunit 2, hybrid-binding domain
WD repeat BOP1/Erb1	DNA-directed RNA polymerase, subunit 2, hybrid-binding domain superfamily
WD40 repeat	Domain of unknown function DUF2183
WD40/YVTN repeat-like-containing domain superfamily	EF-Hand 1, calcium-binding site
WD40-repeat-containing domain	EF-Hand 1, calcium-binding site
WD40-repeat-containing domain superfamily	EF-hand domain
Zinc finger, CCCH-type	EF-hand domain
Zinc finger, CCCH-type superfamily	EF-hand domain pair
	EF-hand domain pair
	Kelch repeat type 1
	Kelch-type beta propeller
	PDZ domain
	PDZ superfamily
	Phosphatidylinositol 3-/4-kinase, catalytic domain
	Phosphatidylinositol 3/4-kinase, conserved site
	Protein kinase-like domain superfamily
	RNA polymerase Rpb2, OB-fold
	RNA polymerase, beta subunit, conserved site
	Tetratricopeptide-like helical domain superfamily
