# Supplementary material for: Ocean acidification at a coastal CO2 vent induces expression of stress-related transcripts and transposable elements in the sea anemone Anemonia viridis
Source: PLoS One. 2019 May 8;14(5):e0210358. doi: 10.1371/journal.pone.0210358 (PMC6505742; doi:10.1371/journal.pone.0210358)
Supplement: S10 Table — The presence of important Symbiodinium sp. stress-response genes in our reference transcriptome assembly have been assessed. Eight different stress-response proteins, corresponding to 62 different proteins from the genus Symbiodinium present in the NCBI database, have been queried to the symbiont reference transcriptome assembly (e-value < 10-3). 29 symbiont contig hits were then BLASTed to the NCBI’s nr database and the best alignments are presented below. The following stress-response genes have been assessed: heat shock proteins 70 and 90 (Hsp70 and Hsp90), superoxide dismutases (SODs), glutathione reductase (GR), thioredoxin (TRX), catalase peroxidase (katG), ascorbate peroxidase (APX) and cytochrome P450 (CYP450). Neither of these proteins was observed as DE in our study. We only observed up-regulation of certain InterPro domains at pH 7.6 compared to normal seawater pH 8.2 (S9 Table). (PDF) [file pone.0210358.s013.pdf]

S10 Table. Presence of symbiont stress-response genes in the reference transcriptome assembly.

Contig ID	Contig length (bp)	Target ID	Protein name	% identity	Alignment length (aa)	e-value
TR102672|c0_g1_i1	383	OLP85464.1	Cytochrome P450 26A1 [Symbiodinium microadriaticum]	52.29	109	8.00E-30
TR1086|c0_g1_i1	1320	OLQ09676.1	Cytochrome c1-2, heme protein, mitochondrial [Symbiodinium microadriaticum]	71.13	291	9.00E-144
TR11222|c3_g9_i3	1227	OLP80221.1	Heat shock 70 kDa protein [Symbiodinium microadriaticum]	94.86	311	0
TR13960|c0_g1_i1	1642	OLQ07063.1	Cytochrome P450 85A [Symbiodinium microadriaticum]	63.61	382	7.00E-178
TR15704|c1_g1_i2	918	AIA81328.1	catalase peroxidase, partial [Symbiodinium sp. A1]	78.67	225	2.00E-129
TR17837|c0_g1_i1	420	OLP97480.1	Cytochrome P450 714A1 [Symbiodinium microadriaticum]	82.76	116	4.00E-52
TR18662|c0_g1_i1	899	OLQ14207.1	L-ascorbate peroxidase 1, cytosolic [Symbiodinium microadriaticum]	89.21	278	9.00E-165
TR19572|c0_g1_i1	575	OLP79028.1	Superoxide dismutase [Mn] [Symbiodinium microadriaticum]	82.49	177	1.00E-78
TR23903|c0_g2_i1	594	OLP84851.1	Cytochrome P450 97B1, chloroplastic [Symbiodinium microadriaticum]	90.26	195	1.00E-115
TR2762|c0_g1_i1	1425	OLP90739.1	putative L-ascorbate peroxidase 3 [Symbiodinium microadriaticum]	60.15	394	6.00E-166
TR28904|c0_g1_i1	1304	OLP85739.1	Cytochrome P450 704C1 [Symbiodinium microadriaticum]	71.43	189	6.00E-160
TR36611|c0_g1_i1	563	OLP91486.1	Glutathione reductase [Symbiodinium microadriaticum]	91.82	159	9.00E-90
TR3661|c2_g10_i2	1709	OLP74754.1	Heat shock protein 90 [Symbiodinium microadriaticum]	93.47	582	0
TR3661|c2_g10_i5	2044	ABA28987.1	heat shock protein 90 3, partial [Symbiodinium sp. C3]	95.83	384	0
TR3661|c2_g11_i3	1304	OLP74754.1	Heat shock protein 90 [Symbiodinium microadriaticum]	97.62	378	0
TR49635|c1_g1_i1	895	OLQ13483.1	Cytochrome P450 86A7 [Symbiodinium microadriaticum]	75.17	298	7.00E-156
TR50638|c1_g1_i1	1968	OLQ13543.1	Cytochrome P450 CYP72A219 [Symbiodinium microadriaticum]	65.98	244	1.00E-162
TR52332|c0_g1_i1	890	OLQ05428.1	Cytochrome P450 704B1 [Symbiodinium microadriaticum]	81.32	257	1.00E-143
TR56472|c2_g2_i1	2083	OLP86233.1	Cytochrome P450 71D11 [Symbiodinium microadriaticum]	87.57	354	0
TR69111|c0_g1_i1	696	OLP85740.1	Cytochrome P450 704B1 [Symbiodinium microadriaticum]	71.14	149	1.00E-85
TR75670|c0_g1_i1	988	OLP96322.1	putative cytochrome P450 120 [Symbiodinium microadriaticum]	68.64	236	8.00E-101

TR8501|c0_g3_i1	1570	OLQ13904.1	Heat shock protein 75 kDa, mitochondrial [Symbiodinium microadriaticum]	90.83	338	0
TR86498|c0_g1_i2	1048	OLP86810.1	Catalase-peroxidase [Symbiodinium microadriaticum]	85.45	220	3.00E-131
TR86969|c0_g1_i1	623	OLP91746.1	Cytochrome P450 144 [Symbiodinium microadriaticum]	73.85	195	4.00E-90
TR93524|c0_g1_i1	456	OLP89004.1	Thioredoxin [Symbiodinium microadriaticum]	71.54	123	4.00E-52
TR94531|c0_g1_i1	368	OLP75262.1	Cytochrome P450 72A11 [Symbiodinium microadriaticum]	61.86	118	2.00E-29
TR95444|c0_g1_i1	768	OLP93090.1	Cytochrome P450 97B3, chloroplastic [Symbiodinium microadriaticum]	83.5	103	1.00E-40
TR95499|c0_g1_i1	272	AHI54358.1	manganese superoxide dismutase, partial [Symbiodinium microadriaticum]	94.37	71	7.00E-41
TR9708|c0_g1_i1	390	OLP86414.1	Thioredoxin [Symbiodinium microadriaticum]	44.74	114	1.00E-15
