# Supplementary material for: Ocean acidification at a coastal CO2 vent induces expression of stress-related transcripts and transposable elements in the sea anemone Anemonia viridis
Source: PLoS One. 2019 May 8;14(5):e0210358. doi: 10.1371/journal.pone.0210358 (PMC6505742; doi:10.1371/journal.pone.0210358)
Supplement: S11 Table — (PDF) [file pone.0210358.s014.pdf]

S11 Table. Differentially expressed SYMBIOTIC transcripts at low seawater pH 7.6 compared to normal seawater pH 8.2 in *Anemonia viridis*.

Our contigs [1]	Kern genes – marked with #[2]	Fold Change	p-value	False Discovery Rate (FDR)	e-value	alignment length	Expressed Sequence Tags[3]	Transcript name - adapted from[2]	expected role - adapted from[2]	Note - adapted from[2]
**TR71702|c0_g1_i1**	#	4.21	3.05E-04	3.39E-02	1.00E-11	33	CL1994Ct1	Mitochondrial fission 1 protein (FIS1)	vesicle	mitochondrial fission
**TR48723|c1_g1_i1**	#	-2.46	1.85E-04	2.45E-02	4.00E-45	93	CL4283Ct1	Carbonic anhydrase 2 cytosolic (CA2-c)	pH regulation/homeostasis	CO2 conversion; pH regulation
**TR34514|c0_g1_i1**	#	-7.19	3.62E-07	3.86E-04	0	448	av01009g10	Uromodulin domain (URO domain)	cell adhesion	cell adhesion/recognition, bacterial defense
**TR21259|c7_g3_i1**		-61.83	1.85E-10	1.23E-06	2.00E-180	658	CL93Ct3	scavenger receptor activity	cell-cell interaction	scavenger receptor activity

[1] Contigs from our reference transcriptome assembly that were found homologous to previously reported transcripts involved in host-symbiotic relationship in *A.viridis*.
[2] *Ganot, P et al. PLoS Genet 2011; 7: e1002187*
[3] *Sabourault, C et al. BMC Genomics 2009; 10: 333*
