# Supplementary material for: Ocean acidification at a coastal CO2 vent induces expression of stress-related transcripts and transposable elements in the sea anemone Anemonia viridis
Source: PLoS One. 2019 May 8;14(5):e0210358. doi: 10.1371/journal.pone.0210358 (PMC6505742; doi:10.1371/journal.pone.0210358)
Supplement: S12 Table — (PDF) [file pone.0210358.s015.pdf]

S12 Table. Differentially expressed APOSYMBIOTIC transcripts at low seawater pH 7.6 compared to normal seawater pH 8.2 in *Anemonia viridis*.

Our contigs [1]	Fold Change	p-value	False Discovery Rate (FDR)	e-value	alignment length	Expressed Sequence Tags[2]	Transcript name - adapted from[3]	expected role - adapted from[2]	Note - adapted from[3]
**TR8883|c0_g4_i2**	6.40	6.65E-05	1.28E-02	0	754	av02093f20	Zinc finger protein 106 homolog (Zfp-106) (Zinc finger protein 474)	DNA damage	Phosphorylated upon DNA damage, probably by ATM or ATR
**TR152|c1_g1_i1**	6.00	2.09E-05	6.07E-03	0	1195	CL535Ct1	Interferon regulatory factor 1 (IRF-1)	txn factor	Interferon regulatory factor 1 (IRF-1)
**TR86900|c0_g1_i1**	4.44	1.17E-04	1.85E-02	0	754	av01041h03	Synaptosomal-associated protein 29 (SNAP-29)	vesicle	
**TR62360|c0_g1_i1**	3.84	1.14E-04	1.83E-02	9.00E-24	55	CL548Ct1	Nucleoside diphosphate kinase B (EC 2.7.4.6)	Metabolism	ATP binding; CTP, GTP and UTP biosynthetic process; magnesium ion binding; nucleoside diphosphate kinase activity
**TR2095|c0_g9_i1**	3.82	1.18E-04	1.87E-02	2.00E-148	393	CL2328Ct1	Heme oxygenase 2 (EC 1.14.99.3) (HO-2)	Heme production	

[1] Contigs from our reference transcriptome assembly that were found homologous to previously reported transcripts involved in host-symbiotic relationship in *A.viridis*.
[2] *Sabourault, C et al. BMC Genomics 2009; 10: 333*
[3] *Ganot, P et al. PLoS Genet 2011; 7: e1002187*
