# Supplementary material for: Ocean acidification at a coastal CO2 vent induces expression of stress-related transcripts and transposable elements in the sea anemone Anemonia viridis
Source: PLoS One. 2019 May 8;14(5):e0210358. doi: 10.1371/journal.pone.0210358 (PMC6505742; doi:10.1371/journal.pone.0210358)
Supplement: S13 Table — Average (S.D.) values of carbonate chemistry parameters at sampling locations off Vulcano Island CO2 seeps. On 13th and 14th May 2013, we performed daily measurements (am and pm) of pHNBS, salinity and temperature (n = 3–4) at the study site during sea anemone sampling, using a 556 MPS YSI (Yellow Springs, USA) probe. The pH sensor was calibrated using NBS scale standards buffers. Three replicate sub-samples of seawater were analyzed at 25° C for total alkalinity (TA) using a titration system (Mettler Toledo, Inc.). (PDF) [file pone.0210358.s016.pdf]

S13 Table. Independent measurements recorded at the sampling site.

	Sampling locations		
	**pH 7.6**	**pH 7.9**	**pH 8.2**
Temperature (°C)	19.1 (0.4)	19.13 (0.4)	19.05 (0.2)
Salinity	38.13 (0.05)	38.1 (0)	38.1 (0)
pH_{NBS}	7.71 (0.3)	7.97 (0.14)	8.17 (0.06)
Total Alkalinity (μmol kg^{-1})	2581	2607	2528
pCO_2 (uatm)	1818 (1201)	819 (286)	437 (69)
CO_2 (μmol kg^{-1})	59 (39)	27 (9)	14 (2)
HCO_3^- (μmol kg^{-1})	2331 (147)	2221 (107)	1987 (53)
CO_3^{2-} (μmol kg^{-1})	104 (61)	160 (44)	223 (22)

The carbonate chemistry parameters were calculated from pH_{NBS}, TA, temperature and salinity with the free-access CO_2 SYS package (*Pierrot, DE and Wallace, DWR. MS Excel Program Developed for CO_2 System Calculations. ORNL/CDIAC-105a. Carbon Dioxide Information Analysis Center, Oak Ridge National Laboratory, U.S. Department of Energy, Oak Ridge, Tennessee (2006)*). using the constants of Roy et al. (*Roy, RN et al., Mar Chem 1993, 4: 249–267*) and Dickson (*Dickson, AG,* J Chem Thermodyn *1990, 22: 113–127*).
