# Supplementary material for: Ocean acidification at a coastal CO2 vent induces expression of stress-related transcripts and transposable elements in the sea anemone Anemonia viridis
Source: PLoS One. 2019 May 8;14(5):e0210358. doi: 10.1371/journal.pone.0210358 (PMC6505742; doi:10.1371/journal.pone.0210358)
Supplement: S14 Table — Adult polyps of the sea anemone were sampled from the individual locations accessed from shore (sampling site pH 7.6 and pH 8.2) or from a boat (pH 7.9). The sampling times at the individual dates could not be synchronized due to bad weather forecast reported for the following days of the stay. Therefore, samples had to be taken as soon as possible on the 14th May. However, we adjusted for the day of sampling in our statistical model (glm edgeR) and found that only small amount of the transcripts were affected by the day of the sampling (see more information in the S4 Table). (PDF) [file pone.0210358.s017.pdf]

S14 Table. Dates and times of sampling of *Anemonia viridis* from the individual sampling locations.

Day of sampling						
	**pH 7.6**		**pH 7.9**		**pH 8.2**	
	**Individuals**	**Sampling time**	**Individuals**	**Sampling time**	**Individuals**	**Sampling time**
13th May 2013						
	pH 7.6 - 1	18:00	pH 7.9 - 1	17:00	pH 8.2 - 1	16:00
	pH 7.6 - 3	18:00	pH 7.9 - 3	17:00	pH 8.2 - 3	16:00
14th May 2013						
	pH 7.6 - 2	12:00	pH 7.9 - 2	11:00	pH 8.2 - 2	10:30
	pH 7.6 - 4	12:00	pH 7.9 - 4	11:00	pH 8.2 - 4	10:30
