# Supplementary material for: Correction: Ocean acidification at a coastal CO2 vent induces expression of stress-related transcripts and transposable elements in the sea anemone Anemonia viridis
Source: PLoS One. 2019 Jun 4;14(6):e0218009. doi: 10.1371/journal.pone.0218009 (PMC6548397; doi:10.1371/journal.pone.0218009)
Supplement: S1 File — (PDF) [file pone.0218009.s001.pdf]

PLOS ONE

RESEARCH ARTICLE

Ocean acidification at a coastal CO_2 vent induces expression of stress-related transcripts and transposable elements in the sea anemone *Anemonia viridis*

Ilona Urbarova[1]*, Sylvain Forêt[2†], Mikael Dahl[1], Åse Emblem[1], Marco Milazzo[3], Jason M. Hall-Spencer[4,5], Steinar D. Johansen[1,6]*

1 Department of Medical Biology, Faculty of Health Sciences, UiT - The Arctic University of Norway, Tromsø, Norway, **2** Evolution, Ecology and Genetics, Research School of Biology, Australian National University, Canberra, ACT, Australia, **3** Department of Earth and Marine Sciences, University of Palermo, Palermo, Italy, **4** School of Biological and Marine Science, University of Plymouth, Plymouth, United Kingdom, **5** Shimoda Marine Research Center, University of Tsukuba, Shimoda City, Shizuoka, Japan, **6** Genomics Research Group, Faculty of Biosciences and Aquaculture, Nord University, Bodø, Norway

† Deceased.
* ilona.urbarova@uit.no (IU); steinar.d.johansen@nord.no (SDJ)

OPEN ACCESS

Citation: Urbarova I, Forêt S, Dahl M, Emblem Å, Milazzo M, Hall-Spencer JM, et al. (2019) Ocean acidification at a coastal CO_2 vent induces expression of stress-related transcripts and transposable elements in the sea anemone *Anemonia viridis*. PLoS ONE 14(5): e0210358. https://doi.org/10.1371/journal.pone.0210358

Editor: Anderson B. Mayfield, Living Oceans Foundation, TAIWAN

Received: July 31, 2018

Accepted: April 5, 2019

Published: May 8, 2019

Copyright: © 2019 Urbarova et al. This is an open access article distributed under the terms of the Creative Commons Attribution License, which permits unrestricted use, distribution, and reproduction in any medium, provided the original author and source are credited.

Data Availability Statement: The RNA-seq raw sequencing data sets of twelve individuals of Anemonia viridis used in this study together with the transcriptome assembly are archived at NCBI's Sequence Read Archive (SRA) under accession number PRJNA448978. The Transcriptome Shotgun Assembly (TSA) has been deposited at DDBJ/EMBL/GenBank under the accession GHCD00000000. The version described in this paper is the first version, GHCD01000000.

Abstract

Ocean acidification threatens to disrupt interactions between organisms throughout marine ecosystems. The diversity of reef-building organisms decreases as seawater CO_2 increases along natural gradients, yet soft-bodied animals, such as sea anemones, are often resilient. We sequenced the polyA-enriched transcriptome of adult sea anemone *Anemonia viridis* and its dinoflagellate symbiont sampled along a natural CO_2 gradient in Italy to assess stress levels in these organisms. We found that about 3.1% of the anemone transcripts, but <1% of the *Symbiodinium* sp. transcripts were differentially expressed. Processes enriched at high seawater CO_2 were linked to cellular stress and inflammation, including significant up-regulation of protective cellular functions and down-regulation of metabolic pathways. Transposable elements were differentially expressed at high seawater CO_2, with an extreme up-regulation (> 100-fold) of the *BEL*-family of long terminal repeat retrotransposons. Seawater acidified by CO_2 generated a significant stress reaction in *A. viridis*, but no bleaching was observed and *Symbiodinium* sp. appeared to be less affected. These observed changes indicate the mechanisms by which *A. viridis* acclimate to survive chronic exposure to ocean acidification conditions. We conclude that many organisms that are common in acidified conditions may nevertheless incur costs due to hypercapnia and/or lowered carbonate saturation states.

Introduction

Reef-forming cnidarians are in global decline due to rapidly increasing levels of atmospheric CO_2 [1], yet non-calcified cnidarians appear to be more resilient [2–6]. Since the Industrial

Funding: This work was supported by grants from the Research Council of Norway (CoralSeq; SDJ), and Tromsø Research Foundation (SDJ). The funders had no role in study design, data collection and analysis, decision to publish, or preparation of the manuscript.

Competing interests: The authors have declared that no competing interests exist.

Revolution, average surface ocean pH has decreased by 0.1 units and is projected to drop by further 0.2–0.4 units by the end of this century, depending on CO_2 emission scenarios [7].

Ocean acidification is expected to lead to widespread marine biodiversity loss and cause major shifts in coastal ecosystems as some species are vulnerable whereas others are more hardy [2, 8]. Many calcifying corals are affected as seawater carbonate saturation levels fall and reefs can be corroded if carbonate levels become undersaturated [9–11]. Some cnidarians grow well in high CO_2 environments, but corrosive waters can weaken the skeletons of those that are calcified and hypercapnia can increase metabolic cost as organisms allocate resources when coping with the changes [12–15]. Surveys in areas with naturally high levels of CO_2 have shown that non-calcified cnidarians (e.g. soft corals, sea anemones and jellyfish) are more resilient to the effects of ocean acidification [3, 5]. However, the molecular mechanisms underlying this resilience are largely unknown.

Physiological measurements of sea anemones exposed to high CO_2 conditions both *in situ* and during laboratory studies show that sea anemones with symbiotic algae increase their primary productivity at high CO_2 due to enhanced carbon availability [3, 16–20]. To better understand the physiological plasticity and adaptive potential of cnidarians to high CO_2, several transcriptome-level studies have been performed, mostly using corals [21–24]. The results of these studies imply that the strongest response is to an acute increase in CO_2 levels [20, 23, 24], and that cnidarians exposed long-term to high CO_2 conditions appear to be more resilient [3, 20, 22, 24]. Although the impact of high CO_2 can be assessed under laboratory conditions, investigations at marine CO_2 seeps are useful for studies of the long-term effects of ocean acidification *in situ* in the whole ecosystem [25], and therefore complement laboratory experiments.

Beyond changes in expression of protein-coding genes, environmental stress can induce the activity of transposable elements that are able to influence both coding and non-coding potential of a genome through genomics rearrangements [26–29]. Transposable elements contribute to the ability to tolerate both biotic and abiotic stresses and could potentially assist the organism to acclimatize or adapt to new environmental conditions [27, 29]. A relatively high proportion of the sea anemone genomes is composed of repeats and transposable elements [30, 31]. However, the involvement of transposable elements in response to stress has not been extensively studied in cnidarians. An increase in transposable element activity upon bleaching in the coral *Montastraea faveolata* is the only observed case so far [32].

As with many organisms that are able to live intertidally, *Anemonia viridis* is physiologically robust and can cope with stressful physicochemical conditions. It has a wider variety of antioxidant enzymes, such as catalases, peroxidases and superoxide dismutases (SOD) than non-symbiotic anemones, and these seem to make this species more resilient to stressors [33]. In addition, the symbiotic cnidarians show an ancient expansion of transposable elements compared to non-symbiotic cnidarians [30], which could contribute in adaptation to a greater range of environmental changes.

The sea anemone *A. viridis* exposed to natural ocean acidification conditions appears to be acclimatized to high CO_2 [3, 18]. The goal of this research was to elucidate the changes in global gene expression patterns in the host and its symbiont that might be associated with the acclimatization of *A. viridis* to high CO_2 conditions *in situ*. We recently reported whole genome and mitochondrial genome sequencing of *A. viridis*, and assessed the expression of small RNAs (miRNAs and piRNAs) and mitochondrial RNAs at *in situ* ocean acidification conditions [34, 35]. Here, we report transcriptomic studies performed on *A. viridis* and its symbiont *Symbiodinium* sp., living at a natural CO_2 gradient off Vulcano Island, Italy, where these organisms are very common in seawater between pH 7.6 and pH 8.2 [3, 18]. We

investigated the transcriptomic response of *A. viridis* living in high seawater CO_2 conditions (pH 7.6) to find out whether they were stressed in the acidified conditions.

Results

Anemonia viridis reference transcriptome

Adult polyps of the sea anemone *A. viridis* were sampled from three different locations along a natural CO_2 gradient near a set of volcanic seeps off Vulcano Island, Sicily (Italy), representing seawater pH conditions 8.2, 7.9, and 7.6 (Fig 1A and 1B). Twelve individual polyps, four from each location, were subjected to total RNA extraction, mRNA isolation and subsequently whole transcriptome sequencing using the Ion Torrent PGM platform (Fig 1C). We generated 3.4 billion nucleotide (nt) sequence data (S1 Table). Sequences obtained from all the individuals were pooled, quality filtered and then used for transcriptome assembly by Trinity [36], yielding 244,294 contigs with an average length of 538 nt (including 41,197 isoforms). Contigs were clustered based on 90% sequence similarity, resulting in 154,015 contigs (including 9,510 isoforms) after filtering for contaminants. About 40% of the contigs had blast hits (38% in nr database and 40% in Swiss-Prot/UniProtKB, e-value $< 10^{-5}$) and 57% of these were annotated with the Blast2GO pipeline B2G4Pipe [37]. This assembly was used as a reference transcriptome in further analyses (see additional assembly metrics in S1 Table).

The reference transcriptome was divided into two fractions using PSyTranS tool (https://github.com/sylvainforet/psytrans) [38], one representing *A. viridis* and the other its symbiotic dinoflagellate *Symbiodinium* sp. (hereafter called the symbiont). This resulted in 90,535 *A. viridis* contigs with average GC-content 41% and 63,480 symbiont contigs with average GC-content 57% (Fig 2 and S1 Table), which were subsequently used in separate transcriptome analyses for each species. The presence of symbiont at all sample locations was confirmed by PCR amplification of the symbiont-specific nuclear apx gene [39] (S2 Table), and from gene expression profiling analysis presented below. We infer that the host-symbiont relationship is not disrupted at low seawater pH conditions.

Anemonia viridis and its symbiont respond differently to low seawater pH

Differential gene expression and its variability among specimens from the same conditions was assessed separately for the host and the symbiont. Quality filtered sequence reads were first aligned to the reference transcriptome, followed by extraction of mapped reads belonging to host and symbiont contigs. We filtered the data set, keeping only transcripts with minimum 10 aligned reads each in at least four samples, and performed differential gene expression analysis accounting for two different variables; pH condition and day of sampling (glm edgeR; S3 Table) [40]. Two specimens appeared to resemble more closely gene expression profiles of individuals from other sampling conditions, and were therefore excluded from further analyses due to greater variability compared to other specimens from the same sampling sites (S3 Table, S1 Fig and Fig 3A). Successive filtering was based on the biological variability and the amount of differentially expressed (DE) transcripts observed among the assessed pH conditions. First, individual 3 from pH 8.2 was filtered away, and subsequently also individual 3 from pH 7.6 was removed from the data set. Only then could we observe a clear separation of individuals based on pH conditions and an increase in the variance explained by PCA bi-plot among the samples (Fig 3A). Because the host/ symbiont contig mapping ratio was found relatively consistent among samples (1.567 ± 0.159), we deemed it unnecessary to normalize our data to this parameter. We found 2832 (~3.1%) of the *A. viridis* transcripts and 519 ($< 1\%$) of the symbiont transcripts to be DE among the assessed pH conditions ($FDR < 0.05$). Nearly all the DE-transcripts appeared to be affected by the pH condition variable only (S4 Table).

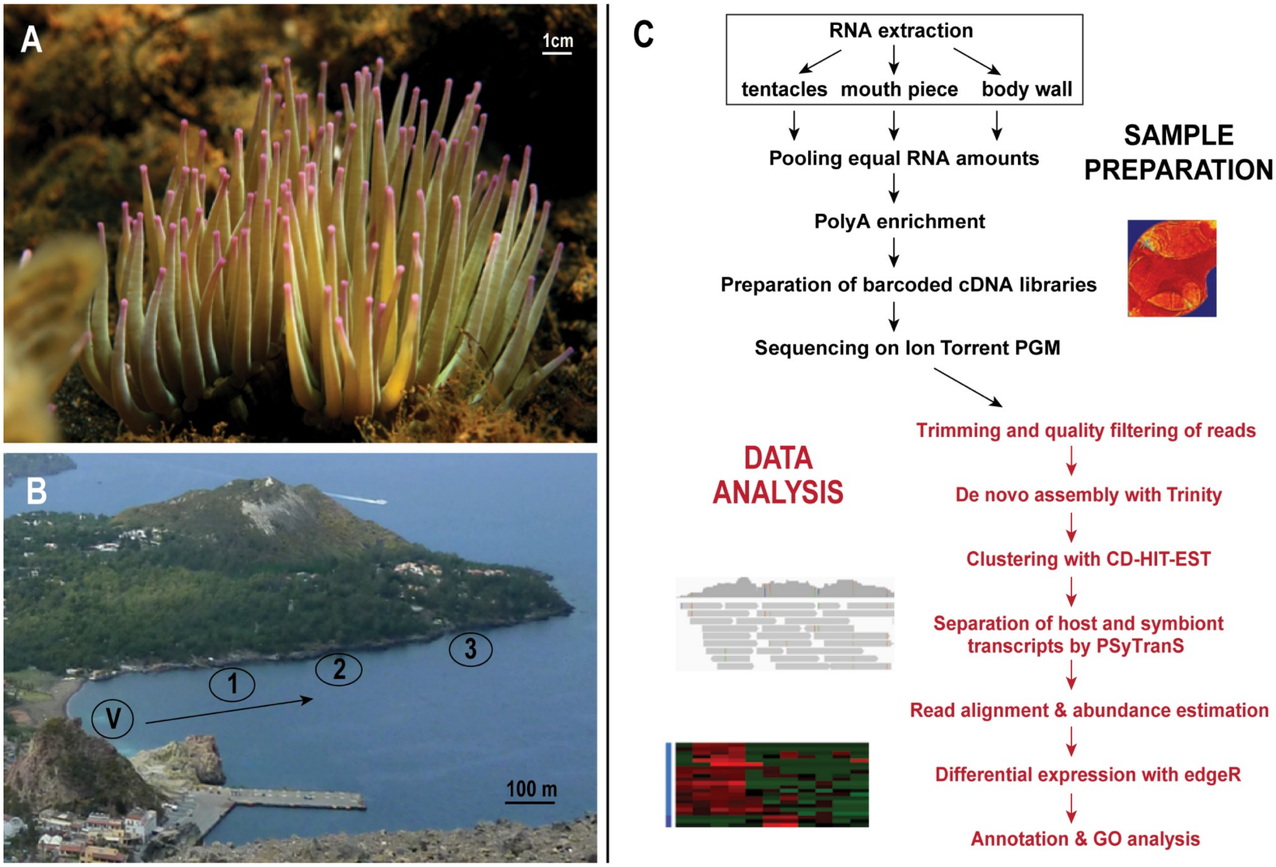

Fig 1. Sampling site overview and data analysis workflow. (A) The sea anemone *Anemonia viridis* growing at high CO_2 (mean pH 7.6) at 1 m depth. (B) Sampling site location at Levante Bay (Vulcano Island, Italy); 1 – low seawater pH site with average pH 7.6; 2 – low seawater pH site with average pH 7.9 and 3 – reference site with average seawater pH 8.2. (C) General overview of laboratory methods and data analyses performed. V – vent site. (Photo credit: A - Demetris Kletou, B - Ilona Urbarova).

https://doi.org/10.1371/journal.pone.0210358.g001

Hierarchical clustering clearly showed that *A*. *viridis* DE-transcripts from specimens at the lowest pH condition studied (pH 7.6) appeared on a separate branch compared to that of pH 7.9 and pH 8.2 (Fig 3B). Clustering of DE-transcripts in the symbiont showed similar results. However, the PCA plot indicated a closer association between symbiont specimens at pH 7.6 and pH 8.2 (Fig 3A). We infer that *A*. *viridis* and the symbiont each responded to pH 7.6 by a different set of DE-transcripts compared to that of pH 7.9 (Fig 3 and S2 Fig).

Down-regulation of transcripts from seawater pH 8.2 to either pH 7.6 or pH 7.9 dominated in *A*. *viridis* and symbiont data sets. *A*. *viridis* showed more transcripts as DE at pH 7.6 compared to pH 7.9, which was contrary to that of the symbiont (Fig 3C). In *A*. *viridis*, we noted 559 up-regulated and 1014 down-regulated transcripts at pH 7.6, and 241 up-regulated and 554 down-regulated transcripts at pH 7.9. In the symbiont however, 83 transcripts were up-regulated and 140 were down-regulated at pH 7.6, versus 59 up-regulated and 208 down-regulated transcripts at pH 7.9. Significant amounts of DE-transcripts in *A*. *viridis* and the symbiont were found to be specific (private) to only one pH condition when compared to pH 8.2 (Fig 3C). From these analyses it therefore appears that low seawater pH has a more significant effect on gene expression in the sea anemone compared to that of the symbiont.

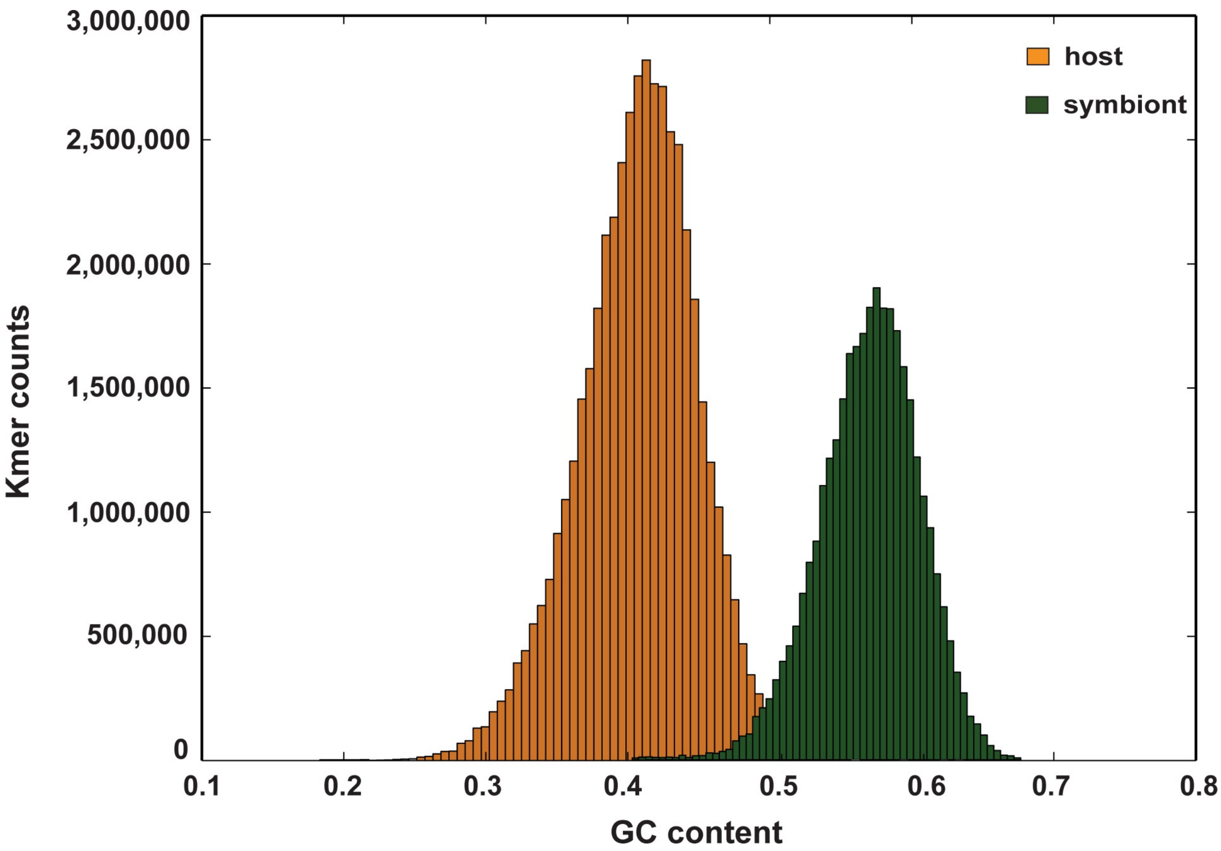

Fig 2. Separation of host and symbiont contigs using PSyTranS software. Host sequences (*Anemonia viridis*) were separated from the symbiont sequences (*Symbiodinium* sp.) using BLAST and Support Vector Machine (SVM) classification implemented in PSyTranS software tool.

https://doi.org/10.1371/journal.pone.0210358.g002

LTR-retrotransposons are heavily up-regulated at low seawater pH

Homology search for transposable elements in our reference transcriptome resulted in 1397 significant contig hits in the RepBase database [41] (1209 in *A*. *viridis* and 188 in the symbiont). The majority (~70%) of these hits showed similarities to known retrotransposons, while DNA transposons were found to constitute only about 30% of all transposons. We found 21 transposable elements (> 200 nt) from 16 *A*. *viridis* contigs as differentially expressed at low seawater pH 7.6 compared to that of normal pH 8.2 conditions (glm edgeR, FDR < 0.05), and most of them were up-regulated (Table 1). Several different transposable element sub-classes were represented, but the most dramatic changes were noted for the long terminal repeat (LTR) retrotransposons. Here, one element of the *BEL*-family showed extreme up-regulation at pH 7.6 (> 100-fold, Table 1). Interestingly, at pH 7.9 only two transposable elements were found to be differentially expressed. LTR retrotransposon (annotated in *Gasterosteus aculeatus*) was four-fold up-regulated and the chicken repeat 1 (*CR1*) element (annotated in *N*. *vectensis*) was two-fold down-regulated. No transposable element could be observed as differentially expressed in the symbiont. We conclude that several classes of transposable elements, and especially the LTR retrotransposons, are strongly activated in *A*. *viridis* at low seawater pH conditions.

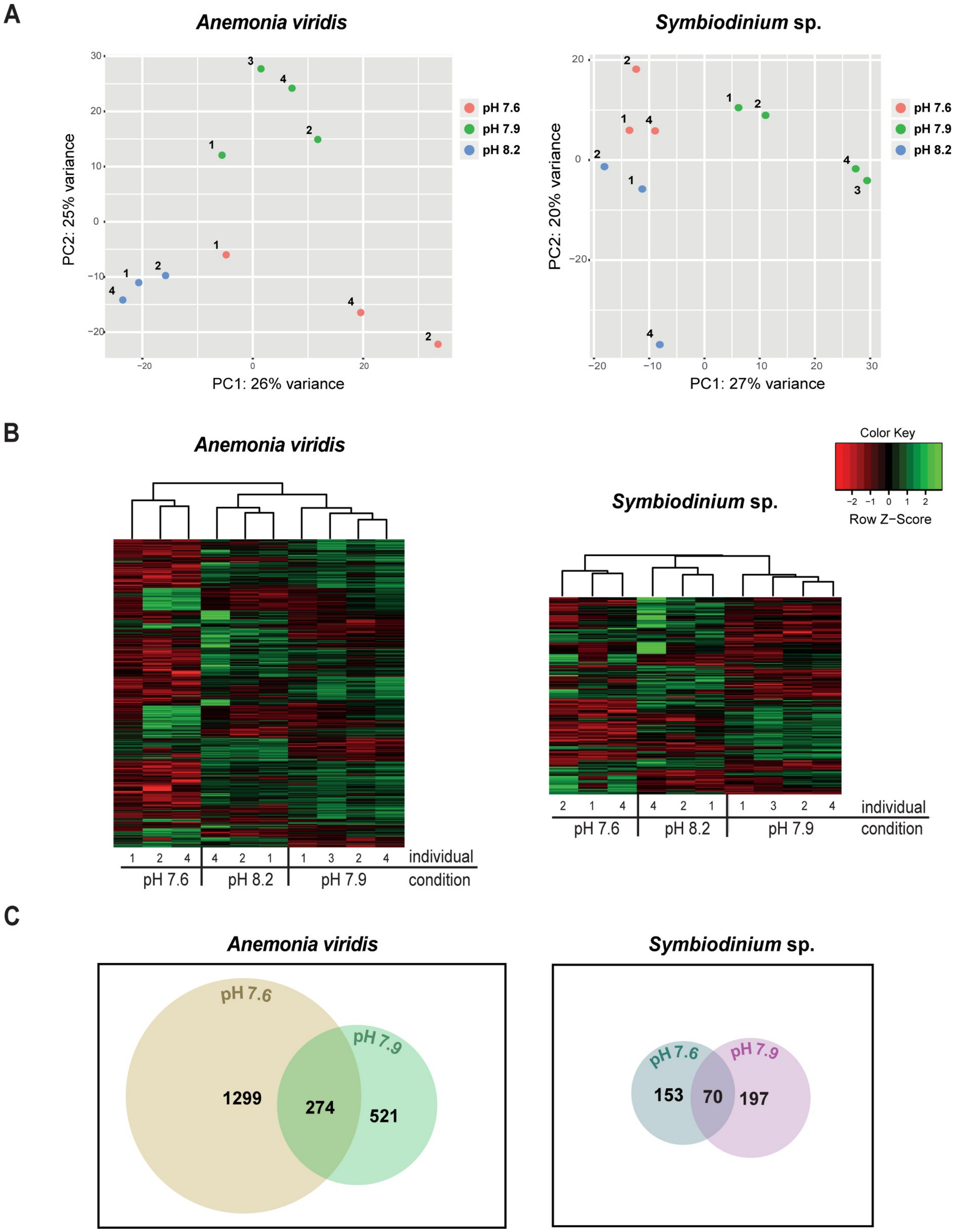

Fig 3. Differential gene expression profiles of *Anemonia viridis* and *Symbiodinium* sp. at the sampling sites. Differential expression (DE) pipeline using a glm edgeR approach was applied to account for both the day of sampling and the different pH where samples were taken. The DE analysis was performed separately for *A. viridis* and *Symbiodinium* sp. (A) Principal component analysis (PCA) plots show clustering similarity of individual samples after specimens 3 from both pH 7.6 and pH 8.2 were filtered off. Numbers in the PCA plots represent different individuals sequenced. (B) Shown is a heatmap with hierarchically clustered, significantly differentially expressed (DE) transcripts between the sampling sites separately for *A. viridis* and *Symbiodinium* sp. (C) Venn diagrams visualize the number of private

and shared DE-transcripts at pH 7.6 and pH 7.9 compared to normal conditions (pH 8.2). *A. viridis* contained 1299 private DE-transcripts at pH 7.6 and 521 private DE-transcripts at pH 7.9. The symbiont contained 153 and 197 private DE-transcripts at pH 7.6 and pH 7.9, respectively. Venn diagrams were created using venneuler in R software.

https://doi.org/10.1371/journal.pone.0210358.g003

Taxonomically restricted genes are mostly down-regulated at low seawater pH

A significant part of our DE-transcript data set included contigs lacking annotation. We found about 63% (1777 DE contigs) for *A. viridis* and 69% (361 DE contigs) for the symbiont with no hits in protein databases (nr and Swiss-Prot/UniProtKB, e-value < 10^{-5}). The great majority

Table 1. Differentially expressed transposable elements (TEs) at pH 7.6 compared to normal pH 8.2 conditions in *Anemonia viridis*.

Transcript	TE region	Fold change	p-value	False Discovery Rate (FDR)	Transposon name	Class of transposon	Specie
TR33113|c0_g2_i1	219–2079	115.06	8.84E-07	3.37E-04	*BEL11-I_AG*	LTR Retrotransposon	*Anopheles gambiae*
TR82105|c3_g1_i1	2757–4658	8.94	1.19E-05	2.00E-03	*Gypsy-43-I_NV*	LTR Retrotransposon	*Nematostella vectensis*
TR15677|c3_g2_i1	1636–2329	8.64	2.11E-06	6.48E-04	*EnSpm-5_CCri*	DNA transposon	*Chondrus crispus*
TR15677|c3_g2_i1	1372–1599	8.61	1.98E-08	1.68E-05	*Copia-33_GM-I*	LTR Retrotransposon	*Glycine max*
TR15677|c3_g2_i1	1177–1326	8.06	6.72E-08	4.48E-05	*Copia-33_GM-I*	LTR Retrotransposon	*Glycine max*
TR82105|c3_g1_i1	3–2337	7.13	7.42E-07	2.93E-04	*Gypsy-21_Adi-I*	LTR Retrotransposon	*Acropora digitifera*
TR8839|c4_g1_i1	815–1093	6.43	1.16E-04	9.04E-03	*TE-X-12_DR*	Nonautonomous TE	*Danio rerio*
TR41995|c0_g2_i1	60–509	4.46	2.41E-05	3.28E-03	*EnSpm-5_CCri*	DNA transposon	*Chondrus crispus*
TR82351|c2_g27_i1	2–301	4.27	1.68E-04	1.14E-02	*BEL1-I_SM*	LTR Retrotransposon	*Schmidtea mediterranea*
TR82351|c2_g40_i1	52–363	4.16	2.13E-04	1.32E-02	*BEL1-I_SM*	LTR Retrotransposon	*Schmidtea mediterranea*
TR82351|c2_g42_i1	1–396	3.92	2.36E-04	1.41E-02	*BEL1-I_SM*	LTR Retrotransposon	*Schmidtea mediterranea*
TR82351|c2_g46_i1	67–366	3.80	2.84E-04	1.61E-02	*BEL1-I_SM*	LTR Retrotransposon	*Schmidtea mediterranea*
TR3718|c11_g21_i4	96–431	3.69	4.04E-04	2.08E-02	*BEL1-I_SM*	LTR Retrotransposon	*Schmidtea mediterranea*
TR41995|c0_g2_i1	642–728	3.57	5.71E-04	2.61E-02	*EnSpm-5_CCri*	DNA transposon	*Chondrus crispus*
TR86841|c5_g2_i1	97–510	3.48	7.07E-04	2.98E-02	*BEL1-I_SM*	LTR Retrotransposon	*Schmidtea mediterranea*
TR2681|c20_g1_i1	1928–2056	2.77	1.39E-03	4.59E-02	*L1M6_5*	Non-LTR Retrotransposon	*Mammalia*
TR2681|c20_g1_i1	2144–2218	2.63	1.47E-03	4.76E-02	*L1M6_5*	Non-LTR Retrotransposon	*Mammalia*
TR33749|c0_g1_i4	282–1017	-4.42	2.94E-04	1.64E-02	*CR1-18_NV*	Non-LTR Retrotransposon	*Nematostella vectensis*
TR68089|c0_g1_i1	88–1078	-21.21	3.29E-04	1.81E-02	*DIRS-1_NV*	LTR Retrotransposon	*Nematostella vectensis*
TR41961|c21_g6_i3	313–784	-22.65	4.00E-06	9.74E-04	*DIRS-35_NV*	LTR Retrotransposon	*Nematostella vectensis*
TR573|c0_g3_i1	2–364	-24.07	7.66E-05	6.80E-03	*DIRS-35_NV*	LTR Retrotransposon	*Nematostella vectensis*

https://doi.org/10.1371/journal.pone.0210358.t001

contained ORFs, but we identified 26 DE contigs for *A*. *viridis* lacking ORFs, and without any hit to protein databases. These may represent putative long non-coding RNAs (lncRNAs) or may originate from untranslated regions (UTRs). We designated the contigs without annotation as taxonomically restricted genes (TRGs), and most of these were found to be down-regulated at low pH conditions in both *A*. *viridis* and the symbiont. For *A*. *viridis*, 56% TRGs were differentially expressed at pH 7.6 compared to pH 8.2, with 69% down-regulated. At pH 7.9, 70% of TRGs were down-regulated. In the symbiont, most TRGs (51%) were differentially expressed at pH 7.9, with 78% down-regulated. Only 143 putative protein domains in 94 TRGs of *A*. *viridis*, and 31 putative protein domains in 20 TRGs for the symbiont were identified by Hidden-Markov model search (hmmscan, e-value $< 10^{-3}$).

Up-regulation of stress-related transcripts and down-regulation of metabolism-related transcripts at low seawater pH

A. *viridis* showed significant up-regulation of stress-related transcripts at low pH, and an increase in oxidative stress response (Table 2). Furthermore, we also noted down-regulation of metabolism-related transcripts at the same conditions. The symbiont showed a similar trend, but only few transcripts could be annotated and therefore results did not appear significant in a gene set enrichment analysis.

A. *viridis* and symbiont contigs with hits in either of the protein databases were annotated with Gene Ontology (GO) terms in order to study biological processes affected by the acidified conditions. Using the GOseq method (S5 Table) [42], a number of GO categories were found over-represented among the DE-transcripts (FDR < 0.05; S6 Table). GOseq terms enriched at pH 7.6 were manually classified into presented ancestor categories (with the most important DE-transcripts shown in Table 2 and extended listing in S7 and S8 Tables). As indicated by hierarchical clustering (Fig 3), the largest expression changes of the *A*. *viridis* transcriptome were found at pH 7.6 compared to pH 7.9 and pH 8.2. GO categories enriched at pH 7.6 were associated with cellular stress, inflammation, and survival (S6 Table). The most significantly up-regulated transcripts were heat shock proteins from Hsp70 and Hsp90 family, molecular chaperones, and other stress response genes (Table 2). We also observed a general down-regulation of transcripts linked to metabolic enzymes. Expression of genes enhancing cell survival was increased, as well as of many gene transcripts leading to NF-κB signalling induction, such as p62 (SQSTM1). Signal transduction pathway members were mostly up-regulated, though transport channels appeared both up- and down-regulated in our analysis. *A*. *viridis* also exhibited a significant inflammatory response at low pH by up-regulation of several immune response genes (Table 2; S7 Table). Repression of global synthesis was supported by up-regulation of EIF2AK3 (also known as PERK), leading to reduction in translation, and by highly up-regulated ubiquitin (S7 Table). In addition, an anti-apoptotic response was observed by up-regulation of Bcl-2-like protein, as also reported in corals by Moya et al. [24], and PRKCI and down-regulation of apoptosis-inducing factor (Table 2). Taken together, these gene features indicated stress-responses at low seawater pH conditions.

For the symbiont, no GO categories appeared significantly over-represented (FDR < 0.05), though the symbiont seemed to respond similarly as the host (S8 Table). Interestingly, we found up-regulation of autophagy 8i transcript at pH 7.6 (S8 Table) and down-regulation of putative chlorophyll A-B binding protein domain by InterPro at both low pH conditions (S9 Table), potentially resulting from oxidative stress response [43]. Despite their successful detection in the reference assembly (S10 Table), the important symbiont stress-response genes (i.e. Hsp70 and 90, SODs, GR, TRX, APX and CYP450) were surprisingly not observed differentially expressed in our study (S8 Table).

Table 2. Selected genes with significant differential expression at low seawater pH 7.6 compared to normal seawater pH 8.2 in *Anemonia viridis*.

Transcripts [1]	Transcript	Fold Change	p-value	False Discovery Rate (FDR)	Transcript length	e-value	Blast similarity [%]
Stress-response genes							
heat shock 70 kda protein (Hsp70)	TR56459|c7_g9_i1	168.49	8.29E-10	1.58E-06	1900	0	89.05
heat shock protein 90 (Hsp90)	TR15677|c3_g2_i1	8.61	1.31E-06	4.52E-04	2783	0	90.40
glucose-regulated protein 78 (Grp78)	TR44825|c0_g1_i1	4.76	2.77E-05	3.76E-03	364	4,96E-15	72.30
glucose-regulated protein 94 (Grp94)	TR41995|c0_g2_i1	4.02	4.37E-05	4.89E-03	2534	0	85.80
hypoxia up-regulated protein 1-like (HYOU1)	TR82131|c1_g7_i3	4.79	3.12E-05	4.05E-03	2474	0	76.35
Antioxidant response							
nuclear factor erythroid 2-related factor (Nrf2)	TR506|c0_g1_i1	5.79	8.62E-06	1.59E-03	2220	1,42E-31	59.90
NF-kappa-b (NF-κB) p100 subunit	TR20187|c0_g1_i1	6.11	1.22E-04	9.65E-03	2584	1,02E-71	56.55
mitogen-activated protein kinase kinase 6 (MAPKK6)	TR18758|c4_g4_i1	3.93	5.68E-04	2.68E-02	1061	0	76.70
superoxide dismutase (CuZnSODb)	TR21605|c0_g1_i2	-3.82	4.07E-04	4.91E-02	490	1,38E-52	76.75
Metabolism							
heme oxygenase 2-like (HMOX2)	TR2095|c0_g3_i1	4.48	6.65E-05	6.48E-03	299	2.90E-43	75.20
hydroxyacid oxidase 1 (HAO1)	TR67672|c1_g7_i9	-5.56	7.37E-06	1.45E-03	794	1,29E-61	72.90
niemann-pick c 2 (Npc2d) – like	TR4599|c1_g6_i3	-6.08	2.01E-04	1.34E-02	818	4,97E-29	55.20
Immune response genes							
interferon regulatory factor 2 (IFN2)	TR152|c1_g1_i1	7.45	3.27E-05	4.16E-03	1320	7,12E-35	69.70
TNF receptor-associated factor 3 (TRAF3)	TR15948|c0_g1_i1	2.88	4.11E-04	2.27E-02	599	2,24E-82	73.10
nucleotide-binding oligomerization domain-2 (NOD2)	TR18769|c1_g4_i7	2.72	3.95E-04	4.82E-02	547	3,99E-06	55.00
Other							
Bcl-2-like 2 protein (BCL2L2)	TR18835|c6_g1_i1	8.39	5.81E-06	1.27E-03	1132	7,01E-58	78.00
eukaryotic translation initiation factor 2-alpha kinase 3 (EIF2AK3)	TR5221|c2_g3_i2	7.30	8.30E-06	1.55E-03	2885	0	52.65
protein kinase C iota type (PRKCI)	TR78358|c3_g1_i1	4.09	3.88E-04	4.69E-02	1462	0	78.85
**sequestosome 1 (p62)**	TR34076|c2_g3_i2	3.71	2.17E-04	1.39E-02	819	7,49E-77	60.50
apoptosis-inducing factor 2 (AIF2)	TR73105|c1_g2_i1	-2.98	5.50E-04	2.62E-02	1503	9,74E-115	64.80

[1] Selection of the most important differentially expressed genes at pH 7.6 compared to pH 8.2 in *A. viridis*. If isoforms exist, the most abundant isoform was selected.

https://doi.org/10.1371/journal.pone.0210358.t002

The expression of genes related to symbiosis is affected at low seawater pH

Many genes involved in innate immunity, lipid metabolism, cell signalling, oxidative stress, apoptosis, autophagy and phagocytosis implicated previously in regulation of host-symbiont relationship in sea anemones [44] appeared differentially expressed at pH 7.6 in our study

(Table 2; S7, S11 and S12 Tables). However, we targeted our analysis on important candidate genes for host-symbiotic relationship maintenance identified specifically in *A. viridis* [39]. To elucidate the involvement of these genes in response to low seawater pH, we performed BLASTN homology search of the DE-transcripts identified in our study to the *A. viridis* EST database [45]. The output was filtered for transcripts found exclusively up-regulated in the symbiotic and the aposymbiotic *A. viridis* [39]. Here, we identified 9 and 15 transcripts significantly differentially expressed at low pH from the symbiotic and aposymbiotic state, respectively (S11 and S12 Tables). The majority of both the symbiotic and the aposymbiotic genes showed down-regulation in our data set. Six of these transcripts were from the 'Kern set' genes (S11 and S12 Tables), including carbonic anhydrases (CA). Significant down-regulation of CA transcripts expression was observed at pH 7.6 compared to pH 8.2 (S11 Table), a result consistent with the findings by Ventura et al. from the same sampling site [20]. Interestingly, we also found a significant down-regulation of Niemann-Pick C2-like protein transcript (specifically its Npc2d variant) both at pH 7.6 (Table 2) and pH 7.9.

Verification of significantly differentially expressed genes by quantitative PCR

In order to verify key findings from the RNA-seq experiment, we re-examined the expression of 13 selected *A. viridis* DE-transcripts involved in stress responses and metabolism by the qPCR approach (S3 Fig). From initial testing of six candidates, we successfully established the ribosomal protein L12 (RPL12), beta-actin and glyceraldehyde 3-phosphate dehydrogenase (GAPDH) transcripts as suitable reference transcripts with relatively stable expression levels between seawater pH 7.6, pH 7.9 and pH 8.2 (S3 Fig). We then examined the expression levels of the selected transcripts from the DE analysis. The main aim was to verify the observed RNA-seq expression profiles of transcripts at pH 7.6 compared to control pH 8.2 by qPCR, as was validated for eight transcripts (Hsp70, NF-kappaB, HMOX2, HYOU1, IRF2, MAPKK6, STOML2 and NPC2d) by ANOVA (S3 Fig and S7 Table). Majority of assessed transcripts did not show significantly differential expression at pH 7.9 compared to pH 8.2 in RNA-seq experiment, as was also confirmed by the qPCR analysis (S3 Fig).

Discussion

Initial observations by Suggett et al. [3] showed that both the abundance of *A. viridis* and the photosynthetic rates of its symbiont (*Symbiodinium* sp. type A19) increased as seawater CO_2 levels rose. There is growing evidence that ocean acidification is a threat to many hard corals, but that their less calcified relatives, e.g. soft corals, jellyfishes and sea anemones, are more resilient [4, 5]. Here, we conducted a transcriptomic response study of the sea anemone and its symbiont. We found high levels of stress-response genes at high CO_2 in the sea anemone, but not in the dinoflagellate. Even though high CO_2 conditions might not be benign or beneficial to some organisms, those organisms may proliferate in hypercapnic conditions because their competitors are less tolerant than they are [46]. At high CO_2, *A. viridis* showed up-regulation of cellular stress response gene transcripts and down-regulation of transcripts involved in metabolism. Furthermore, we observed high up-regulation of several LTR retrotransposons. The symbiont also responded, but fewer genes were differentially expressed. This observation is consistent with previous findings by Barshis et al. [47] and Leggat et al. [48] of symbiotic cnidarians in response to heat stress, where the symbiont was found less affected than the host. Low pH conditions neither appear to represent stressful conditions for the symbiont physiologically. Under normocapnia, the symbiont is apparently inorganic carbon (iC) limited and

the increased iC availability at low pH promotes autotrophy, as discussed by Suggett et al. [3], and in more detail presented in Horwitz et al. [18].

One of our important findings was a dramatic increase in the amount of stress-related transcripts at low pH. A hallmark of cellular stress is up-regulation of heat shock proteins that are involved in many important cellular processes [49]. Specifically, Hsp70 expression has been reported to be induced in response to various environmental and physiological stressors [50]. Furthermore, we observed increased expression of glucose-regulated proteins (GRP; GRP78 and GRP94) at pH 7.6, indicative of endoplasmic reticulum (ER) stress and unfolded protein response (UPR). GRPs assist in folding of damaged proteins, suppress caspase activation, and have positive effects on proliferation [51]. Another hallmark of stress appears linked to autophagy and inflammation. At 0.6 pH units decrease (pH 7.6) we observed a significantly higher expression of SQSTM1, Nrf2, and NF-κB in *A*. *viridis*, all consistent with an up-regulated sequestosome synthesis. In stressful conditions, incorrectly folded proteins accumulate and aggregate, which is then promoted by the ubiquitin-binding protein p62 (also known as SQSTM1; sequestosome 1) [52]. Increased level of p62 activates Nrf2 [53] or NF-κB signalling pathways [54], stimulating inflammatory responses and cellular responses to oxidative injury. Oxidative stress plays apparently a critical role in the cnidarian bleaching cascade by modulating cell death and survival pathways [55, 56]. Respiration, photosynthesis, and autotrophy have previously been reported to increase at low seawater pH in *A*. *viridis* (and its symbiont) at the same sampling site [3, 18]. Rather interestingly, respiration and photosynthetic rates were found unaltered during laboratory-induced short-term ocean acidification conditions in *A*. *viridis* [16]. Surprisingly, we did not observe any significant changes in gene expression of antioxidant enzymes catalase or glutathione S-transferase, and superoxide dismutase CuZnSODb was found to be down-regulated at pH 7.6. However, these observations are in agreement with other studies of symbiotic sea anemones at low pH conditions [39, 44].

Besides observed cellular stress, several metabolism-related enzymes were down-regulated in *A*. *viridis*. We observed a decrease in expression of hydroxyacid oxidase 1 (HAO1) at pH 7.6. HAO1 has previously been associated with oxidative stress responses, suggesting that low gene expression level during oxidative stress provides a mechanism to prevent excessive formation of H_2O_2 [57]. However, we could also find transcripts, such as heme oxygenase 2 (HMOX2) implicated in protection against lipid peroxidation [58], to be up-regulated in *A*. *viridis* at pH 7.6. Importantly, one of the Niemann-Pick type c proteins regulating intracellular sterol-trafficking (NPC2d) was observed down-regulated at pH 7.6 in *A*. *viridis*. NPC2d has been proposed as a key regulator of host-symbiont relationship maintenance [59], localized exclusively in vacuole structures containing the symbionts [59]. The NPC2d gene from *A*. *viridis*, *Exaiptasia pulchella* and *Exaiptasia pallida* have all been observed up-regulated in the symbiotic state, and its expression was negatively affected in sea anemones undergoing various stresses including bleaching [39, 59, 60]. In addition to decrease in NPC2d expression, our data also indicate that the host-symbiont relationship appears affected at high seawater CO_2. However, we observed no physical sign of sea anemone bleaching at the same sampling sites.

One of the most significant findings in our study was the dramatic up-regulation of LTR retrotransposons at high seawater CO_2 (pH 7.6). Activation of retrotransposons, which constitute one of two main classes of transposable genetic elements, may impact the genome structure and genome function [61]. These include genome expansion and chromosomal rearrangements, as well as gene expression abnormalities. Transposable elements appear to play an important biological role in response to biotic and abiotic stresses [27], and an increased activity has been reported during bleaching of the reef building coral *Montastraea faveolata* [32]. Furthermore, substantial activation of LTR retrotransposons has been observed in the marine diatom *Phaeodactylum tricornutum* upon nitrate starvation [62]. But what could

be the biological role of LTR retrotransposon activation in *A. viridis* at high seawater CO_2 conditions? One possibility is that the activation can be a pure selfish response of the transposable element in order to escape from the host environment during stress. This resembles stress-induced proliferations of certain viruses previously reported in corals [63]. Alternatively, the activation could induce genome rearrangements that are of advantage to the host as a response to environmental changes and could lead to local adaptation to the low pH environment. We cannot exclude that some of the observed acclimatization processes involved in response to high CO_2 environment could be influenced by local population genetic structure.

Conclusion

We confirm that the sea anemone *Anemonia viridis* and its algal symbiont *Symbiodinum* sp. are highly resilient to ocean acidification and that these organisms tolerate the changes in carbonate chemistry that occur as pH falls from 8.2 to 7.6. Despite no bleaching of the host, or any other obvious outward signs of stress, at the transcriptome level we observed the following responses in the sea anemone to chronic hypercapnia: 1) up-regulation of protective cellular functions; 2) down-regulation of metabolic pathways; and 3) activation of transposable elements. Our data show that high CO_2 produced a significant stress reaction in *A. viridis*. In contrast, *Symbiodinium* sp. was little affected by increased levels of CO_2 at the transcriptome level. Studies at natural analogues for the effects of ocean acidification have shown that many organisms are unable to cope with long-term elevations in CO_2, but those that do can be abundant. The present study serves as a reminder that so-called 'winners' in a high CO_2 world may incur significant costs.

Materials and methods

Sampling

The temperate sea anemone *Anemonia viridis* (the Snakelocks Anemone) was collected in Levante Bay, Vulcano Island, Sicily - Italy (Fig 1). Here, low seawater pH conditions are created by CO_2 release from a natural vent site at -1 m depth [64, 65]. Sampling was performed on May 13 and 14, 2013, at a depth of 1-2 meters from two different locations >350 m from the vent site along a gradient of decreasing pH (~ pH 7.6 and pH 7.9), and at a reference site ~800 m from the vent site with pH corresponding to ambient seawater levels (~ pH 8.2). For simplicity, we are referring to average pH values throughout this work as reported in Johnson et al. [64], but we know the sea anemones were exposed to variable pH conditions due to shallow currents influenced mostly by dominant western winds, as previously documented [64, 65]. Therefore, we also independently collected measurements of temperature, salinity, pH (NBS scale), and water samples for total alkalinity, at each sampling site to note variability between different days of sampling (S13 Table). In addition, sampling days and times were noted for all the individual samples taken (S14 Table). The sampling site is not only well described in regards to various geochemical measurements (e.g. [64, 65]), several physiological measurements of *A. viridis* have been previously performed at this site [3, 18, 20, 66]. These show that long-term exposure to high pCO_2 has no impact on *Symbiodinium* density (cells mg protein^{-1}), as well as on protein concentrations between sea anemones from the different sampling locations [18, 20, 66]. In addition, the clade of *Symbiodinium* has been previously determined for this sampling site [3]. *Symbiodinum* populations across all sites/ anemones were identified to be the same ITS2 'type' of clade A (A19). A total of twelve *A. viridis* specimens were sampled in two consecutive days (four from each location). Small pieces of tissue (approximately 0.5 cm^3) were collected from body wall, tentacles, and oral disc of each specimen. Each tissue type from each individual was subsequently stored separately at 4˚C in

RNAlater (ThermoFisher Scientific, Waltham, MA, USA), and transported from the sampling site to the laboratory. RNAlater solution was removed and all samples were stored at -80˚C prior to further analysis.

RNA extraction

One ml of ice cold TRIzol reagent (ThermoFisher Scientific, Waltham, MA, USA) was added to the frozen tissue sample after removing excess RNAlater, and then quickly crushed using Precellys lysis homogenizer at 6000 rpm for 30 seconds (Stretton Scientific, Stretton, UK) in order to minimize degradation of RNA. RNA was twice extracted by chloroform, subsequently precipitated in isopropanol at -20˚C overnight, centrifuged at 4˚C, washed with 70% ethanol, and rehydrated in Nuclease-Free Water (ThermoFisher Scientific, Waltham, MA, USA). The RNA quality was examined and RNA amount quantified using the Agilent 2100 Bioanalyzer (Agilent Technologies, Santa Clara, CA, USA) and Qubit 2.0 fluorometer (ThermoFisher Scientific, Waltham, MA, USA), respectively. Total RNA was isolated from each tissue type of an individual separately. Only high quality samples with an RNA integrity number (RIN) value equal to 7 or higher were used in further sequencing experiments.

Whole transcriptome sequencing

Whole transcriptome sequencing of *A*. *viridis* fragment libraries was performed using our in-house Ion Torrent Personal Genome Machine (PGM) sequencing platform (UiT, Norway). Equal amounts of total RNA from three different tissue samples of each specimen were pooled to prepare the sequencing libraries. PolyA enrichment was performed using Dynabeads mRNA DIRECT Purification Kit (ThermoFisher Scientific, Waltham, MA, USA). A total of twelve transcriptome libraries were prepared corresponding to four biological replicates at each location using Ion Total RNA-Seq Kit v2 (ThermoFisher Scientific, Waltham, MA, USA). Here, two replicates were prepared from each sampling day. Libraries were barcoded using Ion Xpress RNA-Seq Barcode 01-16 Kit (ThermoFisher Scientific, Waltham, MA, USA) and were sequenced twice on eight different PGM 318 chips using the Ion PGM 200 Sequencing Kits (ThermoFisher Scientific, Waltham, MA, USA).

Transcriptome assembly and analysis pipeline

Raw Ion Torrent PGM sequencing reads from the same specimens were pooled and trimmed using cutadapt [67]. Barcodes and adapters were removed and reads were quality filtered using Phred quality score > 20. Quality of reads was monitored before, during and after trimming and filtering using the FastQC program (http://www.bioinformatics.babraham.ac.uk/projects/fastqc/). Based on quality graphs from FastQC program, the first 10 nucleotides of each sequence read were removed, and all sequences less than 30 nucleotides were discarded. *De novo* assembly was performed by the Trinity assembler software v2.0.6 using sequences from all individual samples [36]. To reduce contig redundancy, the assembled contigs were further processed by CD-HIT-EST clustering tool using 90% sequence similarity [68]. The assembly was explored for putative contamination by BLASTing against univec and refseq for bacteria and fungi (BLASTN, e-value $< 10^{-5}$), where 352 contigs with higher similarity to these databases than to nucleotide databases of Cnidaria and Dinoflagellata, were filtered away from the assembly. This assembly was used as a reference transcriptome for further analyses. Quality filtered sequence reads from each specimen were aligned separately to the reference transcriptome using BWA mapping software v0.7.12 (BWA-MEM) [69], and an estimation of relative expression levels of transcripts was performed using eXpress v1.5.1 [70]. The assembled reference transcriptome was divided into host and symbiont fraction using BLASTX algorithm for

training data sets and Support Vector Machine (SVM) classification for separating the two fractions; both are implemented in the PSyTranS tool (https://github.com/sylvainforet/psytrans). In this way, all contigs can be assigned to either of the fractions, not only those that have a BLAST match. A total of 27,273 published and predicted protein gene sequences from the sea anemone *N. vectensis* [31] and 47,014 predicted protein gene sequences from the symbiont *Symbiodinium minutum* [71], the closest relatives with published predicted protein sequences at the time of the analysis, were used as training data sets. Successful separation of the host and symbiont contigs was assessed by a GC-content plot, which displayed a clear bimodal distribution. We performed additional analysis to further validate the performance of the PSyTranS tool (S1 Table). After separation of sequence reads, host and symbiont were assessed individually for statistically significant DE-transcripts by the edgeR package [72]. Raw read counts from eXpress were filtered for lowly expressed transcripts (with minimum 10 reads in at least four samples). Data were normalized using TMM (edgeR) [72] and differential gene expression was inferred using a two-factor, negative binomial generalized linear model (glm edgeR) [40], accounting both for the different sampling sites (pH 7.6, 7.9 and 8.2) and various days of sampling (May 13 and 14, 2013). Furthermore, DE-transcripts were filtered with two-fold change difference and a FDR cutoff of 0.05. DE-transcripts were then hierarchically clustered into heatmap based on normalized expression values (rpkm) and scaled by row.

Annotation pipeline

The transcriptome assembly was assessed for homologous protein-coding genes by BLAST (Basic Local Alignment Search Tool) [73]. Contigs were blasted against locally installed protein databases (nr and Swiss-Prot/UniProtKB) on a high-performance cluster using BLASTX (e-value $< 10^{-5}$). Functional GO annotation of our reference transcriptome was carried out using a local Blast2GO pipeline B2G4Pipe [37]. GO analysis was performed using GOseq R package [42] to further explore over- and underrepresented GO categories among different pH conditions. Transcripts with a likely protein-coding capacity were extracted from reference transcriptome by Transdecoder, included in the Trinity package [74], and translated queries were further searched locally for homologous sequences using the hidden Markov model (HMM) [75] against Pfam database [76] (hmmscan, e-value $< 10^{-3}$). InterPro domain search was performed on the Transdecoder output using RunIprScan (http://michaelrthon.com/runiprscan/). Sequences that lacked hits in protein databases were searched for ORF features using OrfPredictor (http://bioinformatics.ysu.edu/tools/OrfPredictor.html) [77].

Identification of transposable elements in the reference transcriptome

Searches for transposable elements in the complete assembly (154,015 contigs) were performed using RepBase database v20.10 [41], which was queried against our reference transcriptome using TBLASTX (e-value $< 10^{-20}$). A tabular output from the search was further run through two perl scripts, "blast92gff3.pl" with additional options -lowscore 0.0001 -alignmax 9999 -exonType exon (http://arthropods.eugenes.org/EvidentialGene/evigene/scripts/blast92gff3.pl.) and the "overbestgene2.pl" (http://iubio.bio.indiana.edu/gmod/tandy/perls/) to create a gff file from blast results and to remove overlapping blast hits, respectively. Only sequence regions corresponding to transposable elements in our transcriptome reference assembly were then extracted from our contigs using BLAST fastacmd tool and substituted the corresponding contigs in the reference transcriptome. Subsequently, an analogous glm edgeR DE analysis was performed, where expression changes of transposable elements were investigated.

Quantitative PCR

Selected transcripts reported by glm edgeR analysis as DE between conditions were further verified by quantitative PCR (qPCR). The ribosomal protein L12 (RPL12), beta-actin and glyceraldehyde 3-phosphate dehydrogenase (GAPDH) were used as endogenous controls for normalization. 20 bp primers with low self-complementarity were designed by Primer 3 [78] to produce 90 – 110 bp products (S2 Table), and amplicons were tested by regular PCR and gel electrophoresis prior to qPCR. PCR products were sequenced by Sanger sequencing to confirm the amplified sequences. RNA for the qPCR analysis was isolated from the same samples that were used for preparation of transcriptome libraries. An equal amount of RNA from each specimen (250 ng) was DNase I treated, and cDNA was subsequently prepared using SuperScript III Reverse Transcriptase (ThermoFisher Scientific, Waltham, MA, USA) with combination of random hexamer primers. cDNA was 10 x diluted and 2 μl was used together with 2x SYBR mix and 2.5 μl of 1 μM primer mix, in 10 μl reactions as input into Roche Light Cycler 96 (Roche, Basel, Switzerland). Each sample was analysed in duplicate by qPCR. Mean Ct (cycle threshold) values for samples were normalized to Ct values of the reference genes (RPL12, beta-actin and GAPDH) and plotted as ΔΔCt values compared to the control condition (pH 8.2).

Supporting information

S1 Fig. Gene expression profiles of *Anemonia viridis* and *Symbiodinium* sp. at low pH. Shown are differential gene expression profiles of (A) the host and (B) the symbiont from two sampling locations of decreasing pH (pH 7.6 and pH 7.9) compared to normal seawater pH 8.2. The differential expression values are plotted as logarithmic fold change (logFC) values, and were calculated from the average expression values of four individuals per sampling location. The heatmap serves only as a visualization of transcripts that are significantly up- and down-regulated at the two low pH conditions compared to normal seawater pH 8.2.
(PDF)

S2 Fig. Verification of significantly differentially expressed genes by quantitative PCR. We assessed the expression of 13 transcripts reported as differentially expressed in our RNA-seq analysis by quantitative (q)PCR. Three individuals per each condition studied (pH 7.6, pH 7.9 and pH 8.2) were examined, and values were normalized to the expression of our 3 reference transcripts: ribosomal protein L12 (RPL12), beta-actin and glyceraldehyde 3-phosphate (GAPDH). Gene expression at two low seawater pH sites (pH 7.6 and pH 7.9) is shown as a relative measure with standard deviations compared to the gene expression at normal seawater pH 8.2.
(PDF)

S3 Fig. Expression levels of six candidate reference genes at the sampling sites. The expression levels of six potential reference transcripts: glyceraldehyde 3-phosphate dehydrogenase (GAPDH), ribosomal protein L12 (RPL12), beta-actin, adenosylhomocysteinase (AHCY), senescence-associated protein and NADH dehydrogenase (NDH), were assessed among the different sampling sites. Presented are the normalized values of these transcripts from the RNA-seq transcript expression matrix. Three transcripts were successfully established as reference transcripts after qPCR testing: GAPDH, RPL12 and beta-actin.
(PDF)

S1 Table. Sequencing information and various metrics of the assembled reference transcriptome of *Anemonia viridis*.
(PDF)

S2 Table. Primer pairs used for regular and quantitative PCR analysis. Shown are primer sequences used for PCR amplification of symbiont-specific nuclear apx gene, together with 15 primer pairs used for verification of results from differential expression analysis by qPCR.
(PDF)

S3 Table. R script for differential expression analyses. Shown is the R script of glm edgeR analyses that was used to find differentially expressed genes between the various pH conditions separately for the host and for the symbiont, using four different individuals per condition. The relative gene expression levels for both the host and the symbiont were similar, hence we infer that the sensitivity of detecting differentially expressed genes in both species was comparable. Additionally, PCA plots and heatmaps were created.
(PDF)

S4 Table. R script to show the influence of each variable in glm edgeR analyses. Shown is the R script used to investigate in detail the influence of each variable on the differential gene expression analyses separately for the host and for the symbiont.
Differentially expressed genes identified in our glm edgeR analysis were separated based on the influence of the two variables in our study, pH condition and day of sampling. By forming contrasts from the design matrix in the glm edgeR pipeline, we were able to identify 2718 DE-transcripts for the host *Anemonia viridis* (95.7%) and 464 DE-transcripts for the symbiont *Symbiodinium* sp. (89.1%) affected by the pH condition variable only. We therefore concluded that day of sampling did not have significant effect on the identification of the differentially expressed genes between the individual pH conditions.
(PDF)

S5 Table. R script for gene set enrichment analyses. Shown is the R script used to find enriched gene ontology (GO) categories at pH 7.6 compared to normal seawater pH 8.2.
(PDF)

S6 Table. Enriched Gene Ontology (GO) categories at pH 7.6. A list of up- and down-regulated gene ontology (GO) categories in *Anemonia viridis* detected at low pH 7.6 compared to normal seawater pH 8.2.
(PDF)

S7 Table. Selected differentially expressed transcripts at low pH compared to normal seawater pH in *A. viridis*. Shown is an extended list of significantly differentially expressed transcripts at low seawater pH 7.6 compared to normal seawater pH 8.2 in *A. viridis* from glm edgeR analysis (FDR < 0.05).
(PDF)

S8 Table. Selected differentially expressed transcripts at low pH compared to normal seawater pH in *Symbiodinium* sp. Shown is a list of selected differentially expressed transcripts at (A) pH 7.6 and (B) pH 7.9 compared to normal seawater pH 8.2 in *Symbiodinium* sp. from glm edgeR analysis (FDR < 0.05).
(PDF)

S9 Table. Analysis of protein signatures in the symbiont DE-data set. Protein-coding regions within differentially expressed transcripts were searched for domains or other functional signatures using InterPro database. Depicted is a list of recognized signatures that appeared up- or down-regulation in the symbiont at low seawater pH 7.6 compared to normal seawater pH 8.2.
(PDF)

S10 Table. Presence of symbiont stress-response genes in the reference transcriptome assembly. The presence of important *Symbiodinium* sp. stress-response genes in our reference transcriptome assembly have been assessed. Eight different stress-response proteins, corresponding to 62 different proteins from the genus *Symbiodinium* present in the NCBI database, have been queried to the symbiont reference transcriptome assembly (e-value $< 10^{-3}$). 29 symbiont contig hits were then BLASTed to the NCBI's nr database and the best alignments are presented below.
The following stress-response genes have been assessed: heat shock proteins 70 and 90 (Hsp70 and Hsp90), superoxide dismutases (SODs), glutathione reductase (GR), thioredoxin (TRX), catalase peroxidase (katG), ascorbate peroxidase (APX) and cytochrome P450 (CYP450). Hsp70, Hsp90, SODs, GR, TRX, APX and CYP450 were not observed as DE in our study. We only observed down-regulation of katG at pH 7.9 (S8 Table) and up-regulation of certain InterPro domains at pH 7.6 (S9 Table).
(PDF)

S11 Table. Differentially expressed SYMBIOTIC transcripts at low seawater pH 7.6 compared to normal seawater pH 8.2 in *Anemonia viridis*.
(PDF)

S12 Table. Differentially expressed APOSYMBIOTIC transcripts at low seawater pH 7.6 compared to normal seawater pH 8.2 in *Anemonia viridis*.
(PDF)

S13 Table. Independent measurements recorded at the sampling site. Average (S.D.) values of carbonate chemistry parameters at sampling locations off Vulcano Island CO_2 seeps. On 13th and 14th May 2013, we performed daily measurements (am and pm) of pH_{NBS}, salinity and temperature (n = 3-4) at the study site during sea anemone sampling, using a 556 MPS YSI (Yellow Springs, USA) probe. The pH sensor was calibrated using NBS scale standards buffers. Three replicate sub-samples of seawater were analyzed at 25° C for total alkalinity (TA) using a titration system (Mettler Toledo, Inc.).
(PDF)

S14 Table. Dates and times of sampling of *Anemonia viridis* from the individual sampling locations. Adult polyps of the sea anemone were sampled from the individual locations accessed from shore (sampling site pH 7.6 and pH 8.2) or from a boat (pH 7.9). The sampling times at the individual dates could not be synchronized due to bad weather forecast reported for the following days of the field trip after first sampling day, 13th May. Therefore, samples had to be taken as soon as possible on the 14th May. However, we adjusted for the day of sampling in our statistical model (glm edgeR) and found that only small amount of the transcripts were affected by the day of the sampling (see more information in the S4 Table).
(PDF)

Acknowledgments

We thank the Notur and Stallo teams (UiT) for assistance in high-performance computing and Dr. Sam Rastrick for providing carbonate chemistry data. We also thank members of the RNA research group at UiT and the Genomics group at Nord University for practical support and discussions.

Author Contributions

Conceptualization: Ilona Urbarova, Jason M. Hall-Spencer, Steinar D. Johansen.

Data curation: Ilona Urbarova, Sylvain Forêt.

Formal analysis: Ilona Urbarova.

Funding acquisition: Steinar D. Johansen.

Investigation: Ilona Urbarova, Steinar D. Johansen.

Methodology: Ilona Urbarova, Sylvain Forêt.

Project administration: Ilona Urbarova, Marco Milazzo, Jason M. Hall-Spencer, Steinar D. Johansen.

Resources: Ilona Urbarova, Sylvain Forêt, Marco Milazzo, Jason M. Hall-Spencer, Steinar D. Johansen.

Software: Sylvain Forêt.

Supervision: Sylvain Forêt, Steinar D. Johansen.

Validation: Ilona Urbarova.

Visualization: Ilona Urbarova, Mikael Dahl, Åse Emblem.

Writing – original draft: Ilona Urbarova, Steinar D. Johansen.

Writing – review & editing: Ilona Urbarova, Sylvain Forêt, Mikael Dahl, Åse Emblem, Marco Milazzo, Jason M. Hall-Spencer, Steinar D. Johansen.

References

1. IPCC. Climate change 2014: Synthesis report. Contribution of working groups I, II and III to the fifth assessment report of the Intergovernmental panel on climate change [Core writing team, R.K. Pachauri and L.A. Meyer (eds.)]. Geneva, Switzerland: IPCC; 2014.
2. Fabricius KE, Langdon C, Uthicke S, Humphrey C, Noonan S, De'ath G, et al. Losers and winners in coral reefs acclimatized to elevated carbon dioxide concentrations. Nat Clim Change. 2011; 1: 165–169.
3. Suggett DJ, Hall-Spencer JM, Rodolfo-Metalpa R, Boatman TG, Payton R, Tye Pettay D, et al. Sea anemones may thrive in a high CO2 world. Glob Chang Biol. 2012; 18: 3015–3025. https://doi.org/10.1111/j.1365-2486.2012.02767.x PMID: 28741826
4. Inoue S, Kayanne H, Yamamoto S, Kurihara H. Spatial community shift from hard to soft corals in acidified water. Nat Clim Change. 2013; 3: 683–687.
5. Hall-Spencer JM, Allen R. The impact of CO2 emissions on 'nuisance' marine species. Rep Biodiv Stud. 2015; 4: 33–46.
6. Horwitz R, Hoogenboom MO, Fine M. Spatial competition dynamics between reef corals under ocean acidification. Sci Rep. 2017; 7: 40288. https://doi.org/10.1038/srep40288 PMID: 28067281
7. Turley C, Gattuso JP. Future biological and ecosystem impacts of ocean acidification and their socio-economic-policy implications. Curr Opin Env Sust. 2012; 4: 278–286.
8. Kroeker KJ, Micheli F, Gambi MC, Martz TR. Divergent ecosystem responses within a benthic marine community to ocean acidification. Proc Natl Acad Sci U S A. 2011; 108: 14515–14520. https://doi.org/10.1073/pnas.1107789108 PMID: 21844331
9. Manzello DP, Kleypas JA, Budd DA, Eakin CM, Glynn PW, Langdon C. Poorly cemented coral reefs of the eastern tropical Pacific: possible insights into reef development in a high-CO2 world. Proc Natl Acad Sci U S A. 2008; 105: 10450–10455. https://doi.org/10.1073/pnas.0712167105 PMID: 18663220
10. Muehllehner N, Langdon C, Venti A, Kadko D. Dynamics of carbonate chemistry, production, and calcification of the Florida Reef Tract (2009-2010): Evidence for seasonal dissolution. Global Biogeochem Cy. 2016; 30: 661–688.
11. Crook ED, Cohen AL, Rebolledo-Vieyra M, Hernandez L, Paytan A. Reduced calcification and lack of acclimatization by coral colonies growing in areas of persistent natural acidification. Proc Natl Acad Sci U S A. 2013; 110: 11044–11049. https://doi.org/10.1073/pnas.1301589110 PMID: 23776217

12. Rodolfo-Metalpa R, Montagna P, Aliani S, Borghini M, Canese S, Hall-Spencer JM, et al. Calcification is not the Achilles' heel of cold-water corals in an acidifying ocean. Glob Chang Biol. 2015; 21: 2238–2248. https://doi.org/10.1111/gcb.12867 PMID: 25641230

13. Hennige SJ, Wicks LC, Kamenos NA, Perna G, Findlay HS, Roberts JM. Hidden impacts of ocean acidification to live and dead coral framework. Proc Biol Sci. 2015; 282: 20150990. https://doi.org/10.1098/rspb.2015.0990 PMID: 26290073

14. Fine M, Tchernov D. Scleractinian coral species survive and recover from decalcification. Science. 2007; 315: 1811. https://doi.org/10.1126/science.1137094 PMID: 17395821

15. Rodolfo-Metalpa R, Houlbreque F, Tambutte E, Boisson F, Baggini C, Patti FP, et al. Coral and mollusc resistance to ocean acidification adversely affected by warming. Nat Clim Change. 2011; 1: 308–312.

16. Jarrold MD, Calosi P, Verberk WCEP, Rastrick SPS, Atfield A, Spicer JI. Physiological plasticity preserves the metabolic relationship of the intertidal non-calcifying anthozoan-Symbiodinium symbiosis under ocean acidification. J Exp Mar Biol Ecol. 2013; 449: 200–206.

17. Hoadley KD, Rollison D, Pettay DT, Warner ME. Differential carbon utilization and asexual reproduction under elevated pCO2 conditions in the model anemone, Exaiptasia pallida, hosting different symbionts. Limnol Oceanogr. 2015; 60: 2108–2120.

18. Horwitz R, Borell EM, Yam R, Shemesh A, Fine M. Natural high pCO2 increases autotrophy in Anemonia viridis (Anthozoa) as revealed from stable isotope (C, N) analysis. Sci Rep. 2015; 5: 8779. https://doi.org/10.1038/srep08779 PMID: 25739995

19. Towanda T, Thuesen EV. Prolonged exposure to elevated CO2 promotes growth of the algal symbiont Symbiodinium muscatinei in the intertidal sea anemone Anthopleura elegantissima. Biol Open. 2012; 1: 615–621. https://doi.org/10.1242/bio.2012521 PMID: 23213455

20. Ventura P, Jarrold MD, Merle PL, Barnay-Verdier S, Zamoum T, Rodolfo-Metalpa R, et al. Resilience to ocean acidification: decreased carbonic anhydrase activity in sea anemones under high pCO2 conditions. Mar Ecol Prog Ser. 2016; 559: 257–263.

21. Davies SW, Marchetti A, Ries JB, Castillo KD. Thermal and pCO2 stress elicit divergent transcriptomic responses in a resilient coral. Front Mar Sci. 2016; 3.

22. Kenkel CD, Moya A, Strahl J, Humphrey C, Bay LK. Functional genomic analysis of corals from natural CO2 -seeps reveals core molecular responses involved in acclimatization to ocean acidification. Glob Chang Biol. 2018; 24: 158–171. https://doi.org/10.1111/gcb.13833 PMID: 28727232

23. Moya A, Huisman L, Ball EE, Hayward DC, Grasso LC, Chua CM, et al. Whole transcriptome analysis of the coral Acropora millepora reveals complex responses to CO2-driven acidification during the initiation of calcification. Mol Ecol. 2012; 21: 2440–2454. https://doi.org/10.1111/j.1365-294X.2012.05554.x PMID: 22490231

24. Moya A, Huisman L, Foret S, Gattuso JP, Hayward DC, Ball EE, et al. Rapid acclimation of juvenile corals to CO2 -mediated acidification by upregulation of heat shock protein and Bcl-2 genes. Mol Ecol. 2015; 24: 438–452. https://doi.org/10.1111/mec.13021 PMID: 25444080

25. Hall-Spencer JM, Rodolfo-Metalpa R, Martin S, Ransome E, Fine M, Turner SM, et al. Volcanic carbon dioxide vents show ecosystem effects of ocean acidification. Nature. 2008; 454: 96–99. https://doi.org/10.1038/nature07051 PMID: 18536730

26. Capy P, Gasperi G, Biemont C, Bazin C. Stress and transposable elements: co-evolution or useful parasites? Heredity (Edinb). 2000; 85 (Pt 2): 101–106.

27. Casacuberta E, Gonzalez J. The impact of transposable elements in environmental adaptation. Mol Ecol. 2013; 22: 1503–1517. https://doi.org/10.1111/mec.12170 PMID: 23293987

28. Fedoroff NV. Presidential address. Transposable elements, epigenetics, and genome evolution. Science. 2012; 338: 758–767. PMID: 23145453

29. McClintock B. The significance of responses of the genome to challenge. Science. 1984; 226: 792–801. PMID: 15739260

30. Baumgarten S, Simakov O, Esherick LY, Liew YJ, Lehnert EM, Michell CT, et al. The genome of Aiptasia, a sea anemone model for coral symbiosis. Proc Natl Acad Sci U S A. 2015; 112: 11893–11898. https://doi.org/10.1073/pnas.1513318112 PMID: 26324906

31. Putnam NH, Srivastava M, Hellsten U, Dirks B, Chapman J, Salamov A, et al. Sea anemone genome reveals ancestral eumetazoan gene repertoire and genomic organization. Science. 2007; 317: 86–94. https://doi.org/10.1126/science.1139158 PMID: 17615350

32. DeSalvo MK, Voolstra CR, Sunagawa S, Schwarz JA, Stillman JH, Coffroth MA, et al. Differential gene expression during thermal stress and bleaching in the Caribbean coral Montastraea faveolata. Mol Ecol. 2008; 17: 3952–3971. https://doi.org/10.1111/j.1365-294X.2008.03879.x PMID: 18662230

33. Richier S, Furla P, Plantivaux A, Merle PL, Allemand D. Symbiosis-induced adaptation to oxidative stress. J Exp Biol. 2005; 208: 277–285. https://doi.org/10.1242/jeb.01368 PMID: 15634847

34. Urbarova I, Patel H, Foret S, Karlsen BO, Jorgensen TE, Hall-Spencer JM, et al. Elucidating the small regulatory RNA repertoire of the sea anemone Anemonia viridis based on whole genome and small RNA sequencing. Genome Biol Evol. 2018; 10: 410–426. https://doi.org/10.1093/gbe/evy003 PMID: 29385567

35. Chi SI, Urbarova I, Johansen SD. Expression of homing endonuclease gene and insertion-like element in sea anemone mitochondrial genomes: Lesson learned from Anemonia viridis. Gene. 2018; 652: 78–86. https://doi.org/10.1016/j.gene.2018.01.067 PMID: 29366757

36. Grabherr MG, Haas BJ, Yassour M, Levin JZ, Thompson DA, Amit I, et al. Full-length transcriptome assembly from RNA-Seq data without a reference genome. Nat Biotechnol. 2011; 29: 644–652. https://doi.org/10.1038/nbt.1883 PMID: 21572440

37. Gotz S, Garcia-Gomez JM, Terol J, Williams TD, Nagaraj SH, Nueda MJ, et al. High-throughput functional annotation and data mining with the Blast2GO suite. Nucleic Acids Res. 2008; 36: 3420–3435. https://doi.org/10.1093/nar/gkn176 PMID: 18445632

38. Bhattacharya D, Agrawal S, Aranda M, Baumgarten S, Belcaid M, Drake JL, et al. Comparative genomics explains the evolutionary success of reef-forming corals. eLife. 2016; 5: e13288. https://doi.org/10.7554/eLife.13288 PMID: 27218454

39. Ganot P, Moya A, Magnone V, Allemand D, Furla P, Sabourault C. Adaptations to endosymbiosis in a cnidarian-dinoflagellate association: differential gene expression and specific gene duplications. PLoS Genet. 2011; 7: e1002187. https://doi.org/10.1371/journal.pgen.1002187 PMID: 21811417

40. McCarthy DJ, Chen Y, Smyth GK. Differential expression analysis of multifactor RNA-Seq experiments with respect to biological variation. Nucleic Acids Res. 2012; 40: 4288–4297. https://doi.org/10.1093/nar/gks042 PMID: 22287627

41. Bao W, Kojima KK, Kohany O. Repbase Update, a database of repetitive elements in eukaryotic genomes. Mob DNA. 2015; 6: 11. https://doi.org/10.1186/s13100-015-0041-9 PMID: 26045719

42. Young MD, Wakefield MJ, Smyth GK, Oshlack A. Gene ontology analysis for RNA-seq: accounting for selection bias. Genome Biol. 2010; 11: R14. https://doi.org/10.1186/gb-2010-11-2-r14 PMID: 20132535

43. Staneloni RJ, Rodriguez-Batiller MJ, Casal JJ. Abscisic acid, high-light, and oxidative stress down-regulate a photosynthetic gene via a promoter motif not involved in phytochrome-mediated transcriptional regulation. Mol Plant. 2008; 1: 75–83. https://doi.org/10.1093/mp/ssm007 PMID: 20031916

44. Meyer E, Weis VM. Study of cnidarian-algal symbiosis in the "omics" age. Biol Bull. 2012; 223: 44–65. https://doi.org/10.1086/BBLv223n1p44 PMID: 22983032

45. Sabourault C, Ganot P, Deleury E, Allemand D, Furla P. Comprehensive EST analysis of the symbiotic sea anemone, Anemonia viridis. BMC Genomics. 2009; 10: 333. https://doi.org/10.1186/1471-2164-10-333 PMID: 19627569

46. Gaylord B, Kroeker KJ, Sunday JM, Anderson KM, Barry JP, Brown NE, et al. Ocean acidification through the lens of ecological theory. Ecology. 2015; 96: 3–15. PMID: 26236884

47. Barshis DJ, Ladner JT, Oliver TA, Palumbi SR. Lineage-specific transcriptional profiles of Symbiodinium spp. unaltered by heat stress in a coral host. Mol Biol Evol. 2014; 31: 1343–1352. https://doi.org/10.1093/molbev/msu107 PMID: 24651035

48. Leggat W, Seneca F, Wasmund K, Ukani L, Yellowlees D, Ainsworth TD. Differential responses of the coral host and their algal symbiont to thermal stress. PLoS One. 2011; 6: e26687. https://doi.org/10.1371/journal.pone.0026687 PMID: 22039532

49. Jolly C, Morimoto RI. Role of the heat shock response and molecular chaperones in oncogenesis and cell death. J Natl Cancer Inst. 2000; 92: 1564–1572. PMID: 11018092

50. Kregel KC. Heat shock proteins: modifying factors in physiological stress responses and acquired thermotolerance. J Appl Physiol (1985). 2002; 92: 2177–2186.

51. Lee AS. Glucose-regulated proteins in cancer: molecular mechanisms and therapeutic potential. Nat Rev Cancer. 2014; 14: 263–276. https://doi.org/10.1038/nrc3701 PMID: 24658275

52. Ma Q. Role of Nrf2 in oxidative stress and toxicity. Annu Rev Pharmacol Toxicol. 2013; 53: 401–426. https://doi.org/10.1146/annurev-pharmtox-011112-140320 PMID: 23294312

53. Nihira K, Miki Y, Ono K, Suzuki T, Sasano H. An inhibition of p62/SQSTM1 caused autophagic cell death of several human carcinoma cells. Cancer Sci. 2014; 105: 568–575. https://doi.org/10.1111/cas.12396 PMID: 24618016

54. Mathew R, Karp CM, Beaudoin B, Vuong N, Chen G, Chen HY, et al. Autophagy suppresses tumorigenesis through elimination of p62. Cell. 2009; 137: 1062–1075. https://doi.org/10.1016/j.cell.2009.03.048 PMID: 19524509

55. Lesser MP. Oxidative stress in marine environments: biochemistry and physiological ecology. Annu Rev Physiol. 2006; 68: 253–278. https://doi.org/10.1146/annurev.physiol.68.040104.110001 PMID: 16460273

56. Weis VM. Cellular mechanisms of Cnidarian bleaching: stress causes the collapse of symbiosis. J Exp Biol. 2008; 211: 3059–3066. https://doi.org/10.1242/jeb.009597 PMID: 18805804

57. Recalcati S, Tacchini L, Alberghini A, Conte D, Cairo G. Oxidative stress-mediated down-regulation of rat hydroxyacid oxidase 1, a liver-specific peroxisomal enzyme. Hepatology. 2003; 38: 1159–1166. https://doi.org/10.1053/jhep.2003.50417 PMID: 14578854

58. Chang EF, Wong RJ, Vreman HJ, Igarashi T, Galo E, Sharp FR, et al. Heme oxygenase-2 protects against lipid peroxidation-mediated cell loss and impaired motor recovery after traumatic brain injury. J Neurosci. 2003; 23: 3689–3696. PMID: 12736340

59. Dani V, Ganot P, Priouzeau F, Furla P, Sabourault C. Are Niemann-Pick type C proteins key players in cnidarian-dinoflagellate endosymbioses? Mol Ecol. 2014; 23: 4527–4540. https://doi.org/10.1111/mec.12876 PMID: 25066219

60. Moya A, Ganot P, Furla P, Sabourault C. The transcriptomic response to thermal stress is immediate, transient and potentiated by ultraviolet radiation in the sea anemone Anemonia viridis. Mol Ecol. 2012; 21: 1158–1174. https://doi.org/10.1111/j.1365-294X.2012.05458.x PMID: 22288383

61. Feschotte C. Transposable elements and the evolution of regulatory networks. Nat Rev Genet. 2008; 9: 397–405. https://doi.org/10.1038/nrg2337 PMID: 18368054

62. Maumus F, Allen AE, Mhiri C, Hu H, Jabbari K, Vardi A, et al. Potential impact of stress activated retrotransposons on genome evolution in a marine diatom. BMC Genomics. 2009; 10: 624. https://doi.org/10.1186/1471-2164-10-624 PMID: 20028555

63. Wilson WH, Dale AL, Davy JE, Davy SK. An enemy within? Observations of virus-like particles in reef corals. Coral Reefs. 2004; 24: 145–148.

64. Johnson VR, Brownlee C, Rickaby REM, Graziano M, Milazzo M, Hall-Spencer JM. Responses of marine benthic microalgae to elevated CO2. Mar Biol. 2013; 160: 1813–1824.

65. Boatta F, D'Alessandro W, Gagliano AL, Liotta M, Milazzo M, Rodolfo-Metalpa R, et al. Geochemical survey of Levante Bay, Vulcano Island (Italy), a natural laboratory for the study of ocean acidification. Mar Pollut Bull. 2013; 73: 485–494. https://doi.org/10.1016/j.marpolbul.2013.01.029 PMID: 23465567

66. Borell EM, Steinke M, Horwitz R, Fine M. Increasing pCO2 correlates with low concentrations of intracellular dimethylsulfoniopropionate in the sea anemone Anemonia viridis. Ecol Evol. 2014; 4: 441–449. https://doi.org/10.1002/ece3.946 PMID: 24634728

67. Martin M. Cutadapt removes adapter sequences from high-throughput sequencing reads. EMBnetjournal. 2011; 17: 3.

68. Li W, Godzik A. Cd-hit: a fast program for clustering and comparing large sets of protein or nucleotide sequences. Bioinformatics. 2006; 22: 1658–1659. https://doi.org/10.1093/bioinformatics/btl158 PMID: 16731699

69. Li H, Durbin R. Fast and accurate short read alignment with Burrows-Wheeler transform. Bioinformatics. 2009; 25: 1754–1760. https://doi.org/10.1093/bioinformatics/btp324 PMID: 19451168

70. Roberts A, Pachter L. Streaming fragment assignment for real-time analysis of sequencing experiments. Nat Methods. 2013; 10: 71–73. https://doi.org/10.1038/nmeth.2251 PMID: 23160280

71. Shoguchi E, Shinzato C, Kawashima T, Gyoja F, Mungpakdee S, Koyanagi R, et al. Draft assembly of the Symbiodinium minutum nuclear genome reveals dinoflagellate gene structure. Curr Biol. 2013; 23: 1399–1408. https://doi.org/10.1016/j.cub.2013.05.062 PMID: 23850284

72. Robinson MD, McCarthy DJ, Smyth GK. edgeR: a Bioconductor package for differential expression analysis of digital gene expression data. Bioinformatics. 2010; 26: 139–140. https://doi.org/10.1093/bioinformatics/btp616 PMID: 19910308

73. Altschul SF, Gish W, Miller W, Myers EW, Lipman DJ. Basic local alignment search tool. J Mol Biol. 1990; 215: 403–410. https://doi.org/10.1016/S0022-2836(05)80360-2 PMID: 2231712

74. Haas BJ, Papanicolaou A, Yassour M, Grabherr M, Blood PD, Bowden J, et al. De novo transcript sequence reconstruction from RNA-seq using the Trinity platform for reference generation and analysis. Nat Protoc. 2013; 8: 1494–1512. https://doi.org/10.1038/nprot.2013.084 PMID: 23845962

75. Eddy SR. Hidden Markov models. Curr Opin Struct Biol. 1996; 6: 361–365. PMID: 8804822

76. Finn RD, Bateman A, Clements J, Coggill P, Eberhardt RY, Eddy SR, et al. Pfam: the protein families database. Nucleic Acids Res. 2014; 42: D222–230. https://doi.org/10.1093/nar/gkt1223 PMID: 24288371

77. Min XJ, Butler G, Storms R, Tsang A. OrfPredictor: predicting protein-coding regions in EST-derived sequences. Nucleic Acids Res. 2005; 33: W677–680. https://doi.org/10.1093/nar/gki394 PMID: 15980561

78. Rozen S, Skaletsky H. Primer3 on the WWW for general users and for biologist programmers. Methods Mol Biol. 2000; 132: 365–386. PMID: 10547847
